# A New Nonlinear Dynamic Speed Controller for a Differential Drive Mobile Robot

**DOI:** 10.3390/e25030514

**Published:** 2023-03-16

**Authors:** Ibrahim A. Hameed, Luay Hashem Abbud, Jaafar Ahmed Abdulsaheb, Ahmad Taher Azar, Mohanad Mezher, Anwar Ja’afar Mohamad Jawad, Wameedh Riyadh Abdul-Adheem, Ibraheem Kasim Ibraheem, Nashwa Ahmad Kamal

**Affiliations:** 1 Department of ICT and Natural Sciences, Norwegian University of Science and Technology, Larsgårdsve-gen, 2, 6009 Ålesund, Norway; 2 Air Conditioning and Refrigeration Techniques Engineering Department, Al-Mustaqbal University College, Hillah 51001, Iraq; 3 Department of Electronics and Communication, College of Engineering, Uruk University, Baghdad 10001, Iraq; 4 College of Computer and Information Sciences, Prince Sultan University, Riyadh 11586, Saudi Arabia; 5 Faculty of Computers and Artificial Intelligence, Benha University, Benha 13518, Egypt; 6 Faculty of Pharmacy, The University of Mashreq, Baghdad 10001, Iraq; 7 Department of Computer Techniques Engineering, Al-Rafidain University College, Baghdad 46036, Iraq; 8 Department of Electrical Engineering, College of Engineering, University of Baghdad, Baghdad 10001, Iraq; 9 Faculty of Engineering, Cairo University, Giza 12613, Egypt

**Keywords:** mobile robot, speed controller, active disturbance rejection control, extended state observer, chattering phenomenon, torque disturbance, system uncertainties

## Abstract

A disturbance/uncertainty estimation and disturbance rejection technique are proposed in this work and verified on a ground two-wheel differential drive mobile robot (DDMR) in the presence of a mismatched disturbance. The offered scheme is the an improved active disturbance rejection control (IADRC) approach-based enhanced dynamic speed controller. To efficiently eliminate the effect produced by the system uncertainties and external torque disturbance on both wheels, the IADRC is adopted, whereby all the torque disturbances and DDMR parameter uncertainties are conglomerated altogether and considered a generalized disturbance. This generalized disturbance is observed and cancelled by a novel nonlinear sliding mode extended state observer (NSMESO) in real-time. Through numerical simulations, various performance indices are measured, with a reduction of 86% and 97% in the *ITAE* index for the right and left wheels, respectively. Finally, these indices validate the efficacy of the proposed dynamic speed controller by almost damping the chattering phenomena and supplying a high insusceptibility in the closed-loop system against torque disturbance.

## 1. Introduction

Generally, in most engineering applications, disturbances/uncertainties (D/Us) are widely presented and negatively affect the performance of the control systems [1]. Control engineering strives to minimize D/Us, and feedforward methods may attenuate or reject the effect of disturbances that can be detected through measurement [2]. Nevertheless, exogenous disturbances cannot be calculated or are exceptionally difficult to calculate. The first spontaneous thought to treat this challenge is to build an observer to estimate the disturbance. Then, an activation signal can be established to compensate for the exogenous disturbance effect. The simplicity of this indication can be expanded to also reject uncertainties. The unmodeled effects of uncertainties or dynamics can be estimated as a proportion of the overall disturbance. As a consequence, a new term was introduced for disturbance activity, which is known as “total disturbance”, which describes the accumulation of exogenous disturbances, unmodeled dynamics, and uncertain conditions in plants. This class of techniques is denoted as estimation and attenuation of disturbance/uncertainty (EAD/U). Several EAD/U structures have been individually suggested. Han first suggested an extended state observer (ESO) in the 1990s [3]. An ESO is generally viewed as playing a major essential role in the technique termed active disturbance rejection control (ADRC) [4]. ADRC consists of three essential parts: a tracking differentiator (TD), extended state observer (ESO), and nonlinear state error feedback controller (NLSEF).

In precision assembly applications, ADRC has been used as a whole configuration; it has been used to perform high-accuracy control of ball screw feed drives [5]. Likewise, a double-loop ADRC scheme was utilized for an active hydraulic suspension system [6]. Taking into account the fact that ADRC is very useful in the field of robotics, this method is particularly useful for the control of quad helicopters because of its capability to handle nonlinear models with significant unsettling influences with vulnerability [7]. Moreover, many engineering systems with ADRC have proven successful [8,9,10]. The main objective of this work is to design a controller that provides an active rejection of the bounded mismatched total disturbances, which have a direct effect on the performance of permanent magnet direct current (PMDC) motors of the DDMR. The controller guarantees a minimum orientation error despite disturbances. Exogenous disturbance involves disturbances including friction torques, fluctuations of the load, changes in parameters for the actuators, and external disturbances that occur due to collisions with obstacles.

The contribution of this paper lies in applying an improved version of Han’s classical ADRC to motion control of a DDMR, which is a nonlinear, multi-input–multi-output (MIMO) system, as an extension of our four previous published papers [11,12,13,14]. The proposed IADRC is constructed by combining three primary units. The first unit is the improved nonlinear tracking differentiator (INTD), which is used to obtain a smooth and accurate differentiation of any nonlinear signal. The INTD also declines signals with frequencies outside a certain frequency band. The second unit in the proposed controller is the improved nonlinear state error feedback (INSEF) controller. This unit is derived by combining the nonlinear gains and the classical PID controller with a new control structure. The last unit is the sliding mode extended state observer (SMESO), which is an expansion of the linear extended state observer (LESO) method; to reduce the chattering in the control signal, the nonlinearity and a sliding mode term are added to the LESO to obtain the proposed SMESO, which performs better than the LESO.

The remainder of this work is structured as follows: Section 2 presents the main results of the IADRC. In Section 3, the convergence of the proposed observers, in addition to stability analysis of the closed-loop system, is investigated. Handling of mismatched disturbances is analyzed within the context of the ADRC in Section 4. Mathematical modeling of the DDMR and PMDC is introduced in Section 5. Section 6 presents the numerical simulations of the proposed IADRC control scheme on DDMR. Finally, the work is concluded in Section 7.

## 2. The Main Results: Improved Active Disturbance Rejection Control (IADRC)

Classical active disturbance rejection control is a powerful controlling method that was first suggested by J. Han [4]. Classical ADRC can be structured by gathering a linear extended state observer (LESO), a tracking differentiator (TD), and a nonlinear state error feedback (NLSEF); the entire structure is presented in [4,15,16].

**Figure 1:**
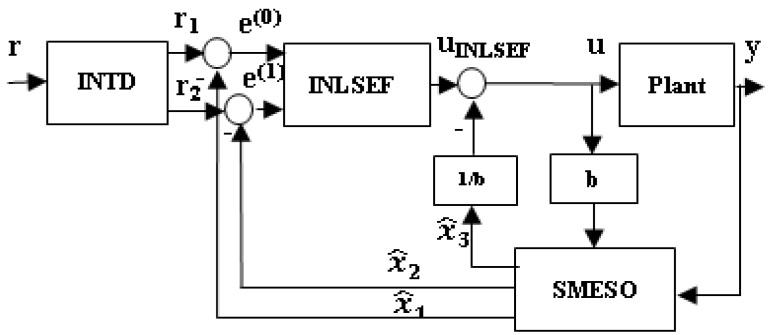
Schematic diagram of the second-order IADRC.

The enhanced configuration of the improved active disturbance rejection control (IADRC) is shown in Figure 1. The following subsections discuss each part of the proposed control scheme supported by necessary explanations.

### 2.1. The Improved Nonlinear TD (INTD)

The *INTD* is the improved version of the classical tracking differentiator. The improvement is achieved by adopting a smooth sigmoid nonlinear function $\varphi \left(.\right)=tanh\left(\cdot \right)$, instead of a $sign\left(\cdot \right)$ function. The reason behind choosing the sigmoid function $tanh\left(\cdot \right)$ is that the $\varphi \left(.\right)=tanh\left(\cdot \right)$ near the origin provides a slope with a smooth shape, which reduces the chattering phenomenon and speeds up the convergence of the proposed tracking differentiator in a significant way. Moreover, adding nonlinearity to the design of the TD increases the robustness of the proposed TD against noise. Another improvement is introduced by integrating nonlinear and linear parts. This TD presents an enhanced dynamic performance relative to Han’s TD. An INTD for second-order systems has been designed using the hyperbolic tangent function [11,17],

(1) $$\left\{\begin{array}{c} {\dot{r}}_{1}=r_{2}\;\\ {\dot{r}}_{2}=-R^{2}\varphi \left(r_{1}\left(t\right)-r\left(t\right)\right)-Rr_{2} \end{array}\right.$$

where $\varphi \left(r_{1}\left(t\right)-r\left(t\right)\right)=\tanh\left(\frac{\beta r_{1}-\left(1-\alpha \right)r}{\gamma }\right)$, r is the reference signal, and *r*_1_ and *r*_2_ are the tracking reference and its derivative, respectively. The coefficients R,β,γ, and α are tuning coefficients, with $0<\alpha \langle 1,\beta \rangle 1,$ γ>0, and R>0. The configuration with the proposed INTD can effectively eliminate the chattering phenomenon and measurement noise and provide swift and smooth tracking of the desired reference signal. To check the stability of the proposed tracking differentiator, the Lyapunov stability approach is utilized [11].

**Definition 1**  (simple sigmoid functions) [18]**.** *a function (*$\varphi :\mathbb{R}\to \left(-1,1\right)$*) is supposed to be a sigmoid. The sigmoid function meets the following conditions:*

1. *The function* $\varphi \left(\cdot \right)$ *is smooth, i.e.,* $\varphi \left(x\right)\in \;C^{\infty }$*;*
2. $\varphi \left(\cdot \right)$ *is an odd function;*
3. *The function* $\varphi \left(\cdot \right)$ *satisfies* $\underset{x\to \pm \infty }{\lim}\left|\varphi \left(x\right)\right|=1$.

**Assumption 1.**  *The function* $\varphi \left(.\right)$ *in definition (4.1) is an odd function with* $\psi \left(y\right)=\int_{0}^{y}\varphi \left(u\right)du\geq 0$*, where u is a variable without any special physical meaning.*

**Figure 2:**
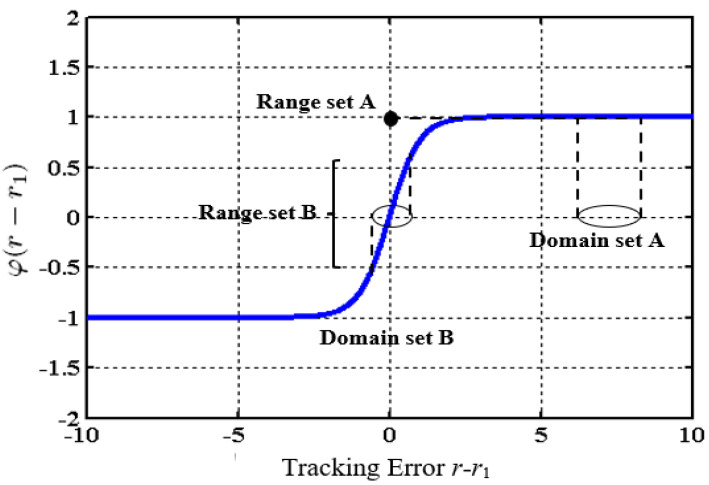
The domain and range sets of the function $\varphi \left(\cdot \right)$

The proposed *INTD* has the following advantages relative to other tracking differentiators:

(i) The proposed tracking differentiator is built using a smooth nonlinear function ($\varphi \left(\cdot \right)$) instead of the $sign\left(\cdot \right)$ function used in most conventional nonlinear differentiators. This is an essential step toward preventing a chattering phenomenon from the output derivatives;
(ii) A second improvement is accomplished by combining the linear and the nonlinear terms. The benefits of this are clear in suppressing high-frequency components in the signal, such as noise. With this feature, the proposed GTD also achieves better performance than other tracking differentiators;
(iii) The saturation feature of the function $\varphi \left(\cdot \right)$ increases the robustness against noisy signals because for large errors, even with a wide range of noise, it is mapped to a small domain set of the function $\varphi \left(\cdot \right)$ (see Figure 2, range and domain sets A);
(iv) Increasing the slope of the continuous function $\varphi \left(\cdot \right)$ near the origin significantly accelerates the convergence of the proposed tracking differentiator (see Figure 2, range and domain sets B).

The convergence of the proposed INTD is investigated in the next theorem.

**Theorem 1.**  *Consider the dynamic system (1). If the signal* $r\left(t\right)$ *is differentiable and* ${sup}_{t\in \left[0,\infty \right)}\left|\dot{r}\left(t\right)\right|=B<\infty$*, then the solution of (1) is convergent in the sense that,* $r_{1}\left(t\right)\;$*is convergent to* $r\left(t\right)$ *as* R→∞.

**Proof.**  Let$,\;t=\frac{\tau }{R}$. Then

(2) $${\dot{r}}_{i}\left(t\right)=\frac{dr_{i}\left(t\right)}{d\tau }\frac{d\tau }{dt}=R\frac{dr_{i}\left(t\right)}{d\tau }\;i\in \left\{1,2\right\}$$

Combining (1) and (2) yields

(3) $$\left\{\;\begin{array}{c} R\frac{dr_{1}\left(\frac{\tau }{R}\right)}{d\tau }=r_{2}\left(\frac{\tau }{R}\right)\;\\ R\frac{dr_{2}\left(\frac{\tau }{R}\right)}{d\tau }=-R^{2}\varphi \left(r_{1}\left(\frac{\tau }{R}\right)-r\left(\frac{\tau }{R}\right)\right)-Rr_{2}\left(\frac{\tau }{R}\right) \end{array}\right.$$

which leads to

(4) $$\left\{\;\begin{array}{c} \frac{dr_{1}\left(\frac{\tau }{R}\right)}{d\tau }=\frac{1}{R}r_{2}\left(\frac{\tau }{R}\right)\;\\ \frac{dr_{2}\left(\frac{\tau }{R}\right)}{d\tau }=-R\varphi \left(r_{1}\left(\frac{\tau }{R}\right)-r\left(\frac{\tau }{R}\right)\right)-r_{2}\left(\frac{\tau }{R}\right) \end{array}\right.$$

Assume

(5) $$\left\{\begin{array}{c} z_{1}\left(\tau \right)=r_{1}\left(\frac{\tau }{R}\right)-r\left(\frac{\tau }{R}\right),\\ z_{2}\left(\tau \right)=\frac{1}{R}r_{2}\left(\frac{\tau }{R}\right)\; \end{array}\right.$$

which results in

(6) $$\left\{\begin{array}{c} \frac{dz_{1}\left(\tau \right)}{d\tau }=\frac{dr_{1}\left(\frac{\tau }{R}\right)}{d\tau }-\frac{dr\left(\frac{\tau }{R}\right)}{d\tau }\\ \frac{dz_{2}\left(\tau \right)}{d\tau }=\frac{1}{R}\frac{dr_{2}\left(\frac{\tau }{R}\right)}{d\tau }\; \end{array}\right.$$

This, together with (4), yields,

(7) $$\left\{\;\begin{array}{c} \frac{dz_{1}\left(\tau \right)}{d\tau }=\frac{1}{R}r_{2}\left(\frac{\tau }{R}\right)-\frac{dr\left(\frac{\tau }{R}\right)}{d\tau },\;\\ \frac{dz_{2}\left(\tau \right)}{d\tau }=\frac{1}{R}\left[-R\varphi \left(r_{1}\left(\frac{\tau }{R}\right)-r\left(\frac{\tau }{R}\right)\right)-r_{2}\left(\frac{\tau }{R}\right)\right]\; \end{array}\right.$$

Then,

(8) $$\left\{\begin{array}{c} {\dot{z}}_{1}\left(\tau \right)=\frac{1}{R}r_{2}\left(\frac{\tau }{R}\right)-\frac{dr\left(\frac{\tau }{R}\right)}{d\tau },\;\\ {\dot{z}}_{2}\left(\tau \right)=-\varphi \left(r_{1}\left(\frac{\tau }{R}\right)-r\left(\frac{\tau }{R}\right)\right)-\frac{1}{R}r_{2}\left(\frac{\tau }{R}\right) \end{array}\right.$$

Substituting (5) and (8), we obtain,

(9) $$\left\{\begin{array}{c} {\dot{z}}_{1}\left(\tau \right)=z_{2}\left(\tau \right)-\frac{dr\left(\frac{\tau }{R}\right)}{d\tau },\;\\ {\dot{z}}_{2}\left(\tau \right)=-\varphi \left(z_{1}\left(\tau \right)\right)-z_{2}\left(\tau \right)\; \end{array}\right.$$

Select the candidate Lyapunov function ($V\left(z\right)$) as

(10) $$V\left(z\right)=\int_{0}^{z_{1}}\varphi \left(v\right)\;dv+\frac{1}{2}z_{2}^{2}\left(\tau \right)$$

The total derivative of $V\left(z\right)$ with respect to τ along the trajectory of the system (9) is given as,

(11) $$\dot{V}\left(z\right)=\varphi \left(z_{1}\right){\dot{z}}_{1}+z_{2}{\dot{z}}_{2}$$

This, together with (8), yields,

(12) $$\dot{V}\left(z\right)=\varphi \left(z_{1}\right)\left[z_{2}\left(\tau \right)-\frac{dr\left(\frac{\tau }{R}\right)}{d\tau }\right]+z_{2}\left[-\varphi \left(z_{1}\left(\tau \right)\right)-z_{2}\left(\tau \right)\right]\;$$

which is derived from

(13) $$\dot{V}\left(z\right)=-\varphi \left(z_{1}\right)\frac{dr\left(\frac{\tau }{R}\right)}{d\tau }-z_{2}^{2}\;$$

Finally, we obtain

(14) $$\dot{V}\left(z\right)\leq \left|\varphi \left(z_{1}\right)\right|\left|\dot{r}\left(t\right)\right|\frac{1}{R}$$

According to Assumption 1 and Definition 1,

(15) $$\dot{V}\left(z\right)\leq \frac{B}{R}$$

(16) $$\underset{R\to \infty }{\lim}\dot{V}\left(z\right)\leq 0$$

Then, the solution of (9) is globally asymptotically stable (GAS) by invoking LaSalle’s invariance principle [19]. It follows that$\;\underset{R\to \infty }{\lim}z_{1}=0$. According to (5), we obtain

(17) $$\underset{R\to \infty }{\lim}r_{1}=r\;$$

□

### 2.2. The Improved Nonlinear State Error Feedback Controller (INSEFC)

Consider the following observable nth-order nonlinear affine-in-control system,

(18) $$\left\{\begin{array}{c} \xi^{\left(n\right)}=f\left(\xi ,\dot{\xi },\ldots ,\xi^{\left(n-1\right)},t\right)+bu\\ y=\xi \; \end{array}\right.$$

where $u\left(t\right)\in C\left(\mathbb{R},\mathbb{R}\right)$ is the control input, $y\left(t\right)\in C\left(\mathbb{R},\mathbb{R}\right)$ is the measured output, b∈ℝ is the input gain, and $f\in C\left({\mathbb{R}}^{n}\times \mathbb{R},\;\mathbb{R}\right)$ is a nonlinear function. It is necessary to design a nonlinear feedback controller (Ψ:ℝ→ℝ) such that the control effort ($u\left(t\right)$) is at its minimum while achieving the following:

1. The closed-loop system is asymptotically stable in the presence of external disturbances, system uncertainties, and measurement noise;
2. The output ($y\left(t\right)$) is forced to track a known reference signal ($r\left(t\right)$), i.e.,
  $\underset{t\to \infty }{\lim}\left|r\left(t\right)-y\left(t\right)\right|=0$, satisfying the transient response specifications;
3. The chattering phenomenon in the control signal ($u\left(t\right)$) is reduced.

**Figure 3:**
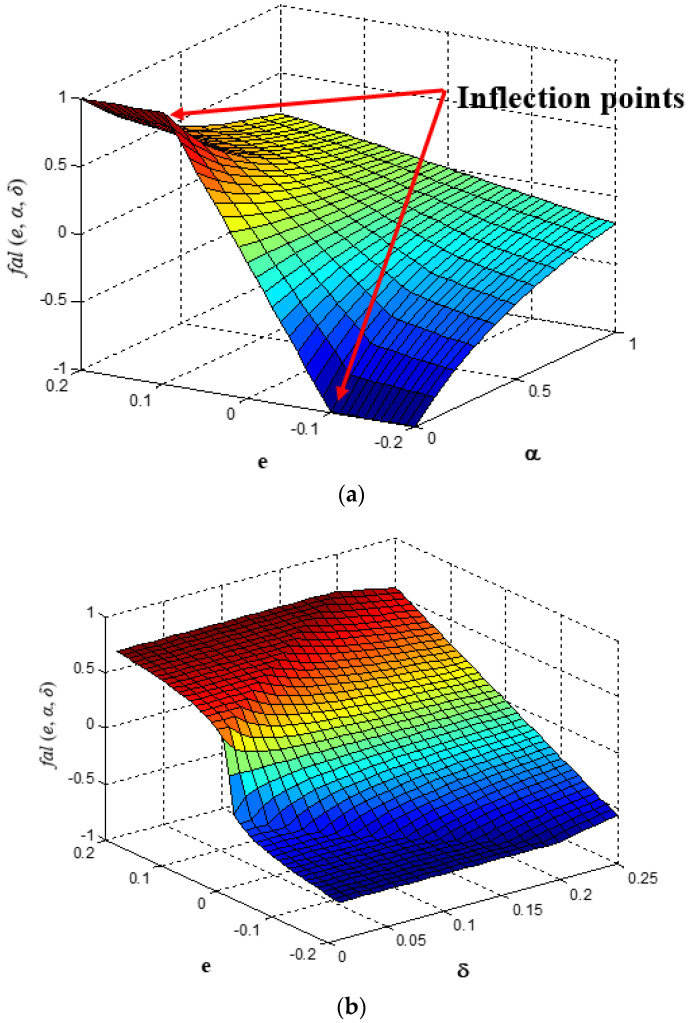
The curve of the $fal\left(\cdot \right)$ function: (**a**) δ=0.1; (**b**) α=0.25.

The original version of the nonlinear state error feedback (SEF) functions in the form of *fal*(.) was first proposed by Han [4] and expressed as,

(19) $$fal\left(e,\alpha ,\delta \right)=\left\{\begin{array}{c} \begin{array}{cc} \frac{e}{\delta^{1-\alpha }}\; & \left|e\right|\leq \delta \end{array}\\ \begin{array}{cc} {\left|e\right|}^{\alpha }sgn\left(e\right) & \left|e\right|>\delta \end{array} \end{array}\right.$$

where δ . is a small number used to express the domain of the linear function near zero [3], and 0<α<1. The $fal\left(\cdot \right)$ is a nonsmooth, piecewise, continuous, nonlinear saturation and a monotonously increasing function [20,21,22,23]. The curve of the $fal\left(\cdot \right)$ function when δ = 0.1 is shown in Figure 3a. The curve of the $fal\left(\cdot \right)\;$function when α = 0.25 is shown in Figure 3b. The $fal\left(\cdot \right)\;$function is nonsmooth at the inflection point [24], and when the value of δ is too small, it is still easy for the phenomenon of high-frequency chattering to appear. This is true even for large δ values [25].

When α = 0.75, the $fal\left(\cdot \right)\;$function is almost linear. In practical terms, the value of α is generally selected as δ = 0.01 [26] and can be further tuned and determined by experiments [27].

The improved nonlinear state error feedback control (INSEFC) law provides more shape flexibility within a wide range of the state error vector. This behavior improves both the performance and the robustness of the controlled system.

The enhanced nonlinear control law uses exponential functions and *sign*(.), and it is established as follows,

(20) $$u_{INLSEF}=\Psi \left(e\right)=k{\left(e\right)}^{T}f\left(e\right)+u_{integrator}$$

where ***e*** is the *n* × 1 state error vector, which is defined as,

(21) $$e={\left[\begin{array}{ccc} e^{\left(0\right)} & \ldots .e^{\left(i\right)}\ldots . & e^{\left(n-1\right)} \end{array}\right]}^{T}$$

where *e*^(*i*)^ is the state error derivative of an *n*th order and expressed as,

(22) $$e^{\left(i\right)}=r_{i+1}-{\hat{\xi }}_{i+1}$$

**Figure 4:**
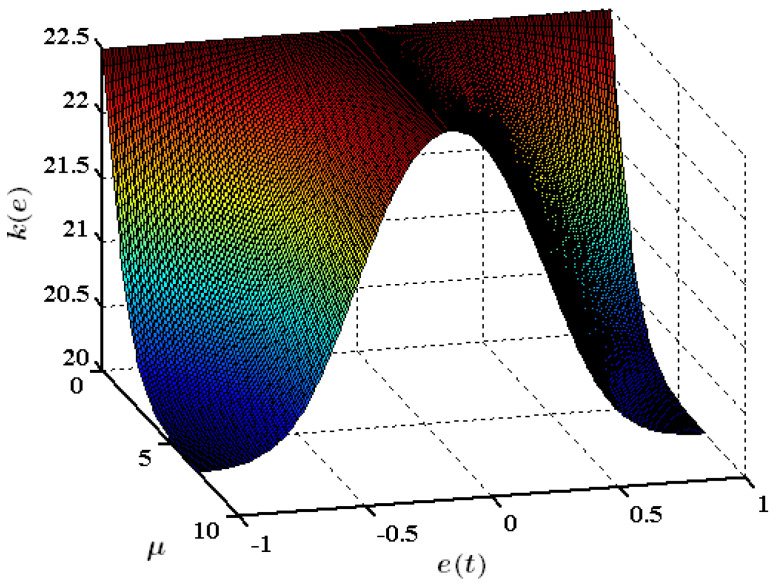
Characteristics of the nonlinear gain function ($k_{i}\left(e\right)$) for $k_{i1}=20$ and $k_{i2}=5$.

*k*(***e***) is a function of nonlinear gains and expressed as,

(23) $$k\left(e\right)=(\begin{array}{c} k_{1}\left(e\right)\\ \vdots \\ k_{i}\left(e\right)\\ \vdots \\ k_{n}\left(e\right) \end{array})=(\begin{array}{c} \left(k_{11}+\frac{k_{12}}{1+\exp\left(\mu_{1}{\left(e^{\left(0\right)}\right)}^{2}\right)}\right)\\ \vdots \\ \begin{array}{c} \left(k_{i1}+\frac{k_{i2}}{1+\exp\left(\mu_{i}{\left(e^{\left(i-1\right)}\right)}^{2}\right)}\right)\\ \begin{array}{c} \vdots \\ \left(k_{n1}+\frac{k_{n2}}{1+\exp\left(\mu_{n}{\left(e^{\left(n-1\right)}\right)}^{2}\right)}\right) \end{array} \end{array} \end{array})$$

where $k_{i1}$,$\;k_{i2}$, and $\mu_{i}$ are positive coefficients, and $i\in \left\{1,2,\ldots ,n\right\}$,. The advantage of $k{\left(e\right)}_{i}$ is that it improves the nonlinear controller’s ability to detect even small errors. When $e^{\left(i-1\right)}$ = 0, $k{\left(e\right)}_{i}=k_{i1}+k_{i2}/2$*,* while as $e^{\left(i-1\right)}$ increases, $k{\left(e\right)}_{i}\approx k_{i1}.$ For values of $e^{\left(i-1\right)}\;$in between, the value of $k{\left(e\right)}_{i}$ lies in the sector of [*k_i*1*_*, *k_i*1*_+k_i*2*_/*2], as shown in Figure 4.

The function *f*(e) is expressed as,

(24) $$f\left(e\right)={\left[\begin{array}{ccc} {\left|e^{\left(0\right)}\right|}^{\alpha_{1}}sign\left(e\right) & \ldots {\left|e^{\left(i\right)}\right|}^{\alpha_{i}}sign\left(e^{\left(i\right)}\right)\ldots & {\left|e^{\left(n-2\right)}\right|}^{\alpha_{n}}sign\left(e^{\left(n-1\right)}\right) \end{array}\right]}^{T}$$

**Figure 5:**
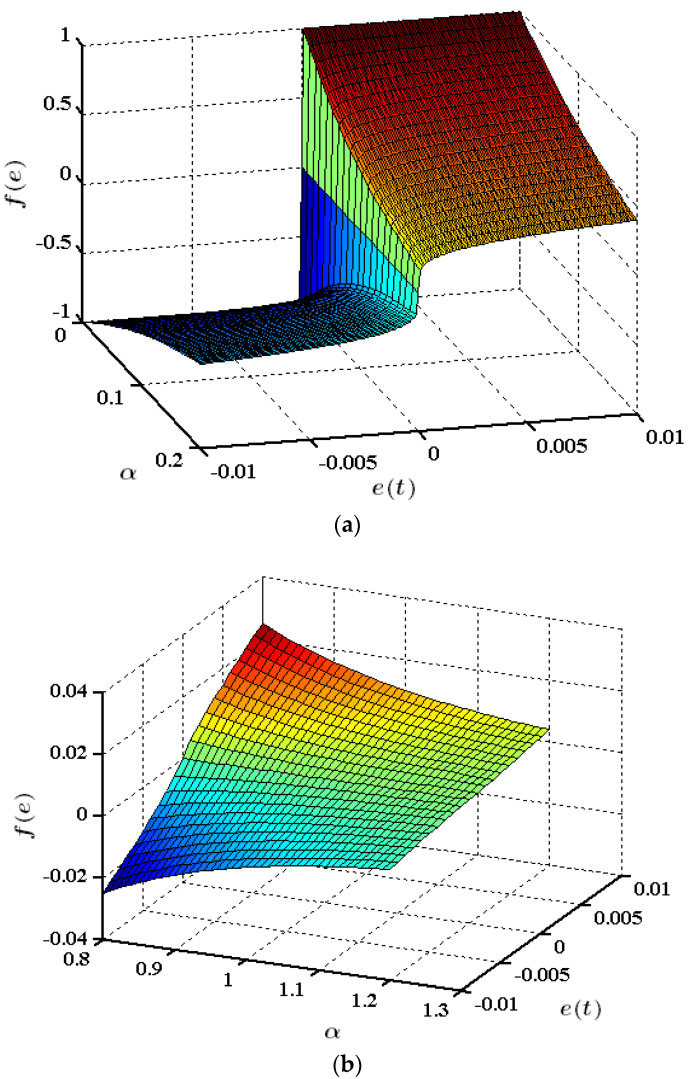
Characteristics of the nonlinear error function ($f\left(e\right)$): $\left(a\right)\;0\leq \alpha \leq 0.2$; (**b**) 0.8≤α≤1.2.

Equation (24) shows significant features in the nonlinear term ${\left|e\right|}^{\alpha }sign\left(e\right)$. For $\alpha_{i}\ll 1$, the term rapidly switches its state, as shown in Figure 5a. This feature makes the error function ($f\left(e\right)$) sensitive to small error values. When α exceeds 1, the nonlinear term becomes less sensitive to small variations in e (see Figure 5b).

The control signal (*u*) can be limited using the nonlinear hyperbolic function ($tanh\left(\cdot \right)$) in the form,

(25) $$u=\delta \;tanh\left(\frac{u_{INLSEF}}{\delta }\right)\;$$

where $u_{INLSEF}$ is defined in (17) and has the following features:

(i) Any real number $\left(-\infty ,\infty \right)$ is mapped to a number in the range of $\left[-\delta ,\delta \right]$;
(ii) The $tanh\left(\cdot \right)$ function is symmetric about the origin, and only zero-valued inputs are mapped to zero outputs;
(iii) The control action (*u*) is limited via mapping but not clipped. Therefore, there are no strong harmonics in the high-frequency range.

**Figure 6:**
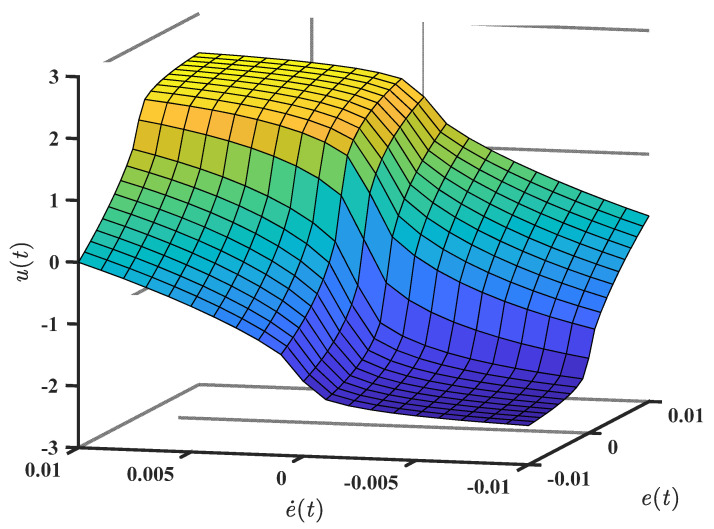
The characteristics of the control signal (u) of (25): n=2, $k_{11}=20,\;k_{12}=5,\;k_{21}=20,\;k_{22}=5,\;\mu_{1}=2.5,\;\mu_{2}=1.5,\;\alpha_{1}=0.5,\;\alpha_{2}=0.5,\;\mathrm{and}\;\delta =2.5$.

Figure 6 shows the control signal (u) against $e\left(t\right)$ and $\dot{e}\left(t\right)$, considering (25).

**Theorem 2.**  *Consider the following observable second-order nonlinear control system (n = 2)*

(26) $$\left\{\begin{array}{c} \ddot{\xi }=f\left(\xi ,\dot{\xi }\right)+bu,\\ y=\xi .\; \end{array}\right.\;$$

**Figure 7:**
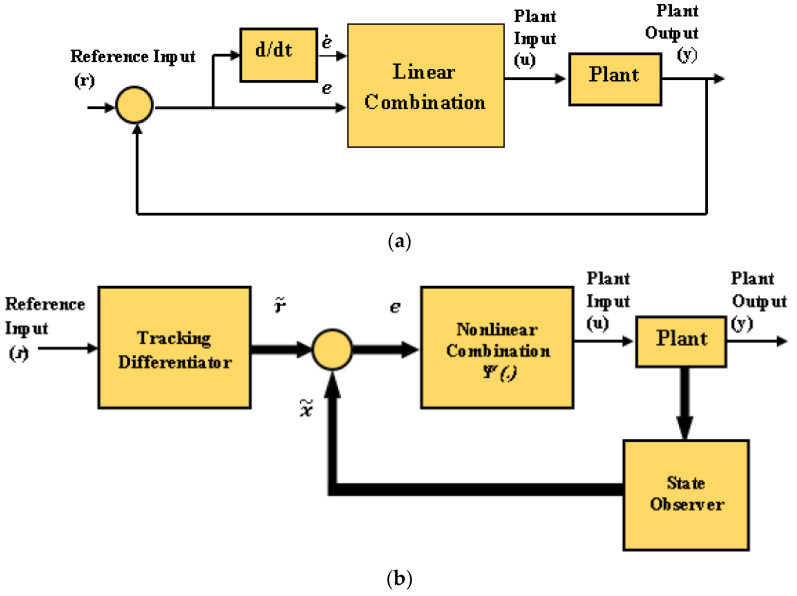
The SISO system in Theorem 1. (**a**) Linear combination control law; (**b**) nonlinear combinational control law.

*as shown in Figure 7a. The PD controller is described as,*

(27) $$u=k_{p}e+k_{d}\dot{e}$$

*where the tracking error is* e=r−y*. Then, the linear control law (u) can be generalized to the form* $u=\Psi \left(e\right)$ *(see Figure 7b) such that* Ψ *is sector-bounded and satisfies* Ψ*(0) = 0.*

**Proof.**  Let$\;x_{1}=x,\;\mathrm{and}\;x_{2}=\dot{x}$. Then, the system (26) can be represented as,

(28) $$\left\{\begin{array}{c} {\dot{\xi }}_{1}=\xi_{2},\;\\ {\dot{\xi }}_{2}=f\left(\xi_{1},\xi_{2}\right)+bu,\\ y=\xi_{1}\; \end{array}\right.$$

Consider a convergent TD, which is described as$\underset{t\to \infty }{\lim}\left|r_{1}-r\right|=0$, $\underset{t\to \infty }{\lim}\left|r_{2}-\dot{r}\right|=0$. Let a convergent state observer be characterized by $\underset{t\to \infty }{\lim}\left|{\tilde{\xi }}_{1}-\xi_{1}\right|=0$, and$\;\underset{t\to \infty }{\lim}\left|{\tilde{\xi }}_{2}-\xi_{2}\right|=0$. Since the tracking error is e=y−r, $\dot{e}=\dot{y}-\dot{r}$; then, the two errors can be defined as $\underset{t\to \infty }{\lim}e=\underset{t\to \infty }{\lim}\left(\xi_{1}-r_{1}\right)$ and$\;\underset{t\to \infty }{\lim}\dot{e}=\underset{t\to \infty }{\lim}\left(\xi_{2}-r_{2}\right)$. Finally, as t→∞, the control law (25) takes the following form: $u=k_{p}(r_{1}-\xi_{1})+k_{d}(r_{2}-\xi_{2})$.

This formula can be expanded for an nth-order system to take the following form: $u=K^{T}e$, where $K={\left(k_{1},k_{2},\ldots ,k_{n}\;\right)}^{T}$ is the gain vector,$\;e={\left(e,\dot{e},\ldots ,e^{\left(n-1\right)}\;\right)}^{T}$ is the tracking error vector, and the linear combination can be generalized to a nonlinear combination formula described as $u=\Psi \left(e\right)$. □

### 2.3. Sliding Mode Extended State Observer (SMESO)

In state space form, the suggested SMESO can be expressed as follows,

(29) $$\dot{\hat{\xi }}=F\hat{X}+B_{1}u+B_{2}g\left(y-{\hat{\xi }}_{1}\right)$$

where $\hat{\xi }\in R^{\left(n+1\right)\times 1}$ is a vector that comprises the observed total disturbance and states of the plant, $\dot{\hat{X}}\in R^{\left(n+1\right)\times 1},B_{1}\in R^{\left(n+1\right)\times 1},B_{2}\in R^{\left(n+1\right)\times 1},\;\mathrm{and}\;F\in R^{\left(n+1\right)\times \left(n+1\right)}$.

$\xi ={\left[\begin{array}{cc} \xi_{1} & \xi_{2\;} \end{array}\begin{array}{cc} \ldots & \xi_{n+1} \end{array}\right]}^{T}$ , $\dot{\hat{\xi }}={\left[\begin{array}{cc} {\dot{\hat{\xi }}}_{1} & {\dot{\hat{\xi }}}_{2\;} \end{array}\begin{array}{cc} \ldots & {\dot{\hat{\xi }}}_{n+1} \end{array}\right]}^{T}$

(30) $$F=\left[\begin{array}{ccc} \begin{array}{cc} 0 & 1\\ 0 & 0 \end{array} & \begin{array}{cc} 0 & 0\\ 1 & 0 \end{array} & \begin{array}{cc} \cdots & 0\\ \cdots & 0 \end{array}\\ \begin{array}{cc} 0 & 0\\ 0 & \vdots \end{array} & \begin{array}{cc} 0 & 1\\ \vdots & \vdots \end{array} & \begin{array}{cc} \cdots & 0\\ \ddots & \vdots \end{array}\\ \begin{array}{cc} 0 & 0\\ 0 & 0 \end{array} & \begin{array}{cc} 0 & 0\\ 0 & 0 \end{array} & \begin{array}{cc} \cdots & 1\\ 0 & 0 \end{array} \end{array}\right]$$

$B_{1}={\left[\begin{array}{ccc} 0 & 0 & \ldots \begin{array}{cc} 1 & 0 \end{array} \end{array}\right]}^{T}$, $B_{2}={\left[\begin{array}{ccc} \beta_{1} & \beta_{2} & \ldots \;\beta_{n+1} \end{array}\right]}^{T}$

Now, $g\left(y-{\hat{\xi }}_{1}\right)=K_{\alpha }{\left|y-{\hat{\xi }}_{1}\right|}^{\alpha }sign\left(y-{\hat{\xi }}_{1}\right)+K_{\beta }{\left|y-{\hat{\xi }}_{1}\right|}^{\beta }\left(y-{\hat{\xi }}_{1}\right)$, where $K_{\alpha }$, α, $K_{\beta }$, and β are appropriate design parameters. With *n =* 2, the SMESO can be expressed as,

(31) $$\{\begin{array}{c} {\dot{\hat{\xi }}}_{1}=x_{2}+\beta_{1}(K_{\alpha }{\left|y-{\hat{\xi }}_{1}\right|}^{\alpha }sign\left(y-{\hat{\xi }}_{1}\right)+K_{\beta }{\left|y-{\hat{\xi }}_{1}\right|}^{\beta }\left(y-{\hat{\xi }}_{1}\right))\\ {\dot{\hat{\xi }}}_{2}=\xi_{3}+bu+\beta_{2}(K_{\alpha }{\left|y-{\hat{\xi }}_{1}\right|}^{\alpha }sign\left(y-{\hat{\xi }}_{1}\right)+K_{\beta }{\left|y-{\hat{\xi }}_{1}\right|}^{\beta }\left(y-{\hat{\xi }}_{1}\right))\\ {\dot{\hat{\xi }}}_{3}=\beta_{3}(K_{\alpha }{\left|y-{\hat{\xi }}_{1}\right|}^{\alpha }sign\left(y-{\hat{\xi }}_{1}\right)+\;K_{\beta }{\left|y-{\hat{\xi }}_{1}\right|}^{\beta }\left(y-{\hat{\xi }}_{1}\right)) \end{array}$$

The SMESO is the nonlinear modified version of the LESO. The proposed SMESO is the third part of the IADRC, which considers the main part that is used to actively estimate what is known as the “total disturbance”. Compared with the LESO, SMESO performs better when it comes to reducing chattering in control signals. In [13], the proposed SMESO demonstrated in detail that estimation error converges to zero asymptotically for nonlinear gain functions. With a sliding term, estimation accuracy is increased for the nonlinear extended state observer. As a result, the proposed method achieves excellent performance when it comes to smoothed control signals, requiring less control energy to accomplish the intended result [13].

## 3. Convergence and Stability Analysis

In this section, the convergence of the proposed SMESO and the stability of the closed-loop system are investigated in detail to validate the proposed design techniques.

### 3.1. Convergence Analysis of the Proposed SMESO

To prove the convergence of the SMESO, the following assumptions are needed.

**Assumption 2.**  *There exists an upper bound for the time derivative of the generalized disturbance (i.e., at least*  $\dot{L}\in C^{1}$ *and* $sup_{t\in \left[0,\infty \right)}\left|\dot{L}\right|=M<\infty$*, where* ∈ℝ*);*

**Assumption 3.**  *L is a continuously differentiable function;*

**Assumption 4.**  $V:{\mathbb{R}}^{n+1}\to {\mathbb{R}}^{+}$ *and* $W:{\mathbb{R}}^{n+1}\to {\mathbb{R}}^{+}$ *are continuously differentiable functions with [16],*

(32) $$\lambda_{1}{\left\|\eta \right\|}^{2}\leq V\left(\eta \right)\leq \lambda_{2}{\left\|\eta \right\|}^{2}\;,\;W\left(\eta \right)={\left\|\eta \right\|}^{2}\;$$

(33) $$\sum_{i=1}^{n-1}\frac{\partial V\left(\eta \right)}{\eta_{i}}\left(\eta_{i+1}-a_{i}k\left(\frac{\eta_{1}}{\omega_{0}{}^{\rho }}\right).\eta_{1}\right)-\frac{\partial V\left(\eta \right)}{\partial y_{n}}a_{n}k\left(\frac{\eta_{1}}{\omega_{0}{}^{n}}\right)\eta_{1}\leq -W\left(\eta \right)$$

**Theorem 3.**  (SMESO convergence)**.** *Given the system of (18) and SMESO of (29), it follows that under assumptions A3 and A5, for any initial conditions,*

(i) $\underset{t\to \infty }{\lim}\left|\xi_{i}\left(t\right)-{\hat{\xi }}_{i}\left(t\right)\right|=O\left(\frac{1}{\omega_{0}{}^{n+2-i}}\right)$
(ii) $\underset{\begin{array}{c} t\to \infty \\ \omega_{0}\to \infty \end{array}}{\lim}\left|\xi_{i}\left(t\right)-{\hat{\xi }}_{i}\left(t\right)\right|=0$

*where* $\xi_{i}$ *and* ${\hat{\xi }}_{i}$ *symbolize the state of (18) and (29), respectively, where* $i\in \left\{1,2,\ldots ,n+1\right\}$.

**Proof.**  Let $e_{i}=\xi_{i}-{\hat{\xi }}_{i}$, $i\in \left\{1,2,\ldots ,n+1\right\}$. Correspondingly, let

(34) $$\eta_{i}=\omega_{0}{}^{n-i}e_{i}\left(\frac{t}{\omega_{0}}\right)\;,\;i\in \left\{1,2,\ldots ,n+1\right\}$$

Then, the dynamics of the estimation error can be expressed in a time scale as,

(35) $$\left\{\begin{array}{c} \begin{array}{c} \frac{d\eta_{1}}{dt}=\eta_{2}-a_{1}k\left(\frac{\eta_{1}}{\omega_{0}{}^{n-1}}\right)\eta_{1}\;\\ \frac{d\eta_{2}}{dt}=\eta_{3}-a_{2}k\left(\frac{\eta_{1}}{\omega_{0}{}^{n-1}}\right)\eta_{1}\;\\ \vdots \; \end{array}\\ \begin{array}{c} \frac{d\eta_{n}}{dt}=\eta_{n}-a_{n}k\left(\frac{\eta_{1}}{\omega_{0}{}^{n-1}}\right)\eta_{1}\;\\ \frac{d\eta_{n+1}}{dt}=\frac{\Delta_{h}}{\omega_{0}{}^{2}}-a_{n+1}k\left(\frac{\eta_{1}}{\omega_{0}{}^{n-1}}\right)\eta_{1}\; \end{array} \end{array}\right.$$

Let the candidate Lyapunov functions ($V,W:{\mathbb{R}}^{n+1}\to {\mathbb{R}}^{+}$) denoted by $V\left(\eta \right)=\langle P\eta ,\eta \rangle =\eta^{T}P\eta$, where $\eta \in {\mathbb{R}}^{n+1}$, and P is a positive definite symmetric matrix. Consider (22) of assumption A4 with $\lambda_{1}=\lambda_{min}\left(P\right)$ and $\lambda_{2}=\lambda_{max}\left(P\right)$, where $\lambda_{min}\left(P\right)$ and $\lambda_{max}\left(P\right)$ are the minimum and maximum eigenvalues of P, respectively.$\;\dot{V}$ with regard to t over η (over the solution of (35) )is determined as follows:

(36) $${\left.\dot{V}\left(\eta \right)\right|}_{along\;\left(35\right)}=\sum_{i=1}^{n+1}\frac{\partial V\left(\eta \right)}{\partial \eta_{i}}{\dot{\eta }}_{i}\left(t\right)$$

Then,

(37) $${\left.\dot{V}\left(\eta \right)\right|}_{along\;\left(35\right)}=\overset{n-1}{\underset{i=1}{\sum }}\frac{\partial V\left(\eta \right)}{\eta_{i}}\left(\eta_{i+1}\left(t\right)-a_{i}k\left(\frac{\eta_{1}\left(t\right)}{\omega_{0}{}^{n}}\right).\eta_{1}\left(t\right)\right)-\frac{\partial V\left(\eta \right)}{\partial \eta_{n}}a_{n}k\left(\frac{\eta_{1}\left(t\right)}{\omega_{0}{}^{n}}\right).\eta_{1}\left(t\right)+\frac{\partial V\left(\eta \right)}{\partial \eta_{n+1}}\frac{M}{\omega_{0}{}^{2}}$$

Consider (33) of assumption A4; then,

(38) $${\left.\dot{V}\left(\eta \right)\right|}_{along\;\left(35\right)\;}\leq -W\left(\eta \right)+\frac{\partial V\left(\eta \right)}{\partial \eta_{n+1}}\frac{M}{\omega_{0}{}^{2}}$$

As $V\left(\eta \right)\leq \lambda_{max}\left(P\right){\left\|\eta \right\|}^{2}$ and $\left|\frac{\partial V\left(\eta \right)}{\partial \eta_{n+1}}\right|\leq \|\frac{\partial V\left(\eta \right)}{\partial \eta }\|$, then $\left|\frac{\partial V}{\partial \eta_{n+1}}\right|\leq 2\lambda_{max}\left(P\right)\|\eta \|$. As $V\left(\eta \right)\leq \lambda_{max}\left(P\right){\left\|\eta \right\|}^{2}=\lambda_{max}\left(P\right)W\left(\eta \right)$. Thus, $-W\left(\eta \right)\leq -\frac{V\left(\eta \right)}{\lambda_{max}\left(\;P\right)}$. Finally, because $\lambda_{min}\left(P\right){\left\|\eta \right\|}^{2}\leq V\left(\eta \right)$, this leads to $\|\eta \|\leq \sqrt{\frac{V\left(\eta \right)}{\lambda_{min}\left(P\right)}}$. Accordingly, and given assumption A4, $\dot{V}\left(\eta \right)$ becomes,

$\dot{V}\left(\eta \right)\leq -\frac{V\left(\eta \right)}{\lambda_{max}\left(P\right)}+\frac{M}{\omega_{0}{}^{2}}2\lambda_{max}\left(P\right)\frac{\sqrt{V\left(\eta \right)}}{\sqrt{\lambda_{min}\left(P\right)}}$. Since $\frac{d}{dt}\sqrt{V\left(\eta \right)}=\frac{1}{2}\frac{1}{\sqrt{V\left(\eta \right)}}\dot{V}\left(\eta \right)$, then,

(39) $$\frac{d}{dt}\sqrt{V\left(\eta \right)}\leq \frac{1}{2}\frac{1}{\sqrt{V\left(\eta \right)}}\left(-\frac{V\left(\eta \right)}{\lambda_{max}\left(P\right)}+\frac{M}{\omega_{0}{}^{2}}2\lambda_{max}\left(P\right)\frac{\sqrt{V\left(\eta \right)}}{\sqrt{\lambda_{min}\left(\eta \right)}}\right)$$

which gives

(40) $$\frac{d}{dt}\sqrt{V\left(\eta \right)}\leq -\frac{\sqrt{V\left(\eta \right)}}{2\lambda_{max}\left(P\right)}+\frac{M}{\omega_{0}{}^{2}}\frac{\lambda_{max}\left(P\right)}{\sqrt{\lambda_{min}\left(P\right)}}\;$$

which can be solved as

$$\sqrt{V\left(\eta \right)}\leq \frac{2M\lambda_{max}^{2}\left(P\right)}{\omega_{0}{}^{2}\sqrt{\lambda_{min}\left(P\right)}}\left(1-e^{-\frac{t}{2\lambda_{max}\left(P\right)}}\right)+\sqrt{V\left(\eta \left(0\right)\right)}e^{-\frac{t}{2\lambda_{max}\left(P\right)}}$$

According to assumption A4, we have $\lambda_{min}\left(P\right){\left\|\eta \right\|}^{2}\leq V\left(\eta \right)$. This leads to $\|\eta \|\leq \sqrt{\frac{V\left(\eta \right)}{\lambda_{min}\left(P\right)}}$. Then,

$$\|\eta \|\leq \sqrt{\frac{1}{\lambda_{min}\left(P\right)}}\;\left(\frac{2M\lambda_{max}^{2}\left(P\right)}{\omega_{0}{}^{2}\sqrt{\lambda_{min}\left(P\right)}}\left(1-e^{-\frac{t}{2\lambda_{max}\left(P\right)}}\right)+\sqrt{V\left(\eta \left(0\right)\right)}e^{-\frac{t}{2\lambda_{max}\left(P\right)}}\right)$$

which yields

(41) $$\|\eta \|\leq \;\frac{2M\lambda_{max}^{2}\left(P\right)}{\omega_{0}{}^{2}\lambda_{min}\left(P\right)}\left(1-e^{-\frac{t}{2\lambda_{max}\left(P\right)}}\right)+\sqrt{\frac{V\left(\eta \left(0\right)\right)}{\lambda_{min}\left(P\right)}}e^{-\frac{t}{2\lambda_{max}\left(P\right)}}$$

It follows from (34) that,

$$\left|\xi_{i}-{\hat{\xi }}_{i}\right|\leq \frac{1}{\omega_{0}{}^{n-i}}\|\eta \left(\omega_{0}t\right)\|$$

It follows from (41) that,

$$\left|\xi_{i}-{\hat{\xi }}_{i}\right|\leq \frac{1}{\omega_{0}{}^{n-i}}\left(\frac{2M\lambda_{max}^{2}\left(P\right)}{\omega_{0}{}^{2}\lambda_{min}\left(P\right)}\left(1-e^{-\frac{\omega_{0}t}{2\lambda_{max}\left(P\right)}}\right)+\sqrt{\frac{V\left(\eta \left(0\right)\right)}{\lambda_{min}\left(P\right)}}e^{-\frac{\omega_{0}t}{2\lambda_{max}\left(P\right)}}\right)$$

Finally,

(42) $$\underset{t\to \infty }{\lim}\left|\xi_{i}-{\hat{\xi }}_{i}\right|=\frac{1}{\omega_{0}{}^{n+2-i}}\frac{2M\lambda_{max}^{2}\left(P\right)}{\lambda_{min}\left(P\right)}=O\left(\frac{1}{\omega_{0}{}^{n+2-i}}\right)\;$$

and

(43) $$\underset{\begin{array}{c} t\to \infty \\ \omega_{0}\to \infty \end{array}}{\lim}\left|\xi_{i}-{\hat{\xi }}_{i}\right|$$

□

### 3.2. Stability Analysis of the Closed-Loop System

In this section, the closed-loop stability is investigated for a general nonlinear SISO uncertain system with an ADRC controller.

**Assumption 5.**  *The states* ${\hat{\xi }}_{i}$ $\left(i=1,2,\ldots ,n\right)$ *and the generalized disturbance* $\xi_{n+1}=f$ *of an* n*-dimensional uncertain nonlinear SISO system are estimated by a convergent ESO, which produces the estimated states* ${\hat{\xi }}_{i},\;i\in \left\{1,2,\ldots ,n\right\}\;$*of the plant and the estimated generalized disturbance* ${\hat{\xi }}_{n+1}\;$*as* t→∞*, i.e.,*

(44) $$\underset{t\to \infty }{\lim}\left|\xi_{i}-{\hat{\xi }}_{i}\right|=0,\;i\in \left\{1,2,\ldots ,n\right\},\;$$

and

(45) $$\underset{t\to \infty }{\lim}\left|f-{\hat{\xi }}_{n+1}\right|=0$$

**Assumption 6.**  *A tracking differentiator produces a trajectory (*$r_{i}\;,\;i\in \left\{1,2,\ldots ,n\right\}$*) with minimum set point change. The trajectory converges to a reference trajectory (*$r^{\left(i-1\right)}$*) for*$i\in \left\{1,2,\ldots ,n\right\}$ *with* $r^{\left(n\right)}=0$ *as* t→∞*, i.e.,*

(46) $$\underset{t\to \infty }{\lim}\left|r^{\left(i-1\right)}-r_{i}\right|=0,\;i\in \left\{1,2,\ldots ,n\right\}$$

**Theorem 4.**  *Consider a* n*-dimensional uncertain nonlinear SISO system expressed as*

(47) $$\left\{\begin{array}{c} \xi_{i}=\xi_{i+1},\;i\in \left\{1,2.\ldots ,n-1\right\}\;\\ \begin{array}{c} {\dot{\xi }}_{n}=f\left(\xi_{1},\xi_{2},\ldots ,\xi_{\rho },w,t\right)+u\;\\ y=\xi_{1}\; \end{array} \end{array}\right.$$

*The system (47) is controlled by the linearization control law (LCL) signal (*u*) expressed by,*

(48) $$u=v-{\hat{\xi }}_{n+1}$$

*where* v *is expressed by,*

(49) $$v={𝓀}_{1}\left({\tilde{e}}_{1}\right){\tilde{e}}_{1}+{𝓀}_{2}\left({\tilde{e}}_{2}\right){\tilde{e}}_{2}+\ldots +{𝓀}_{n}\left({\tilde{e}}_{n}\right){\tilde{e}}_{n}$$

*where* ${\tilde{e}}_{i}=r_{i}-{\hat{\xi }}_{i}\;,i\in \left\{1,2,\ldots ,n\right\}$ *is the tracking error, and* ${𝓀}_{i}:\;\mathbb{R}\to {\mathbb{R}}^{+},\;i\in \left\{1,2,\ldots ,n\right\}$*; assume that assumptions A5 and A6 hold. Then*

(50) $$\underset{t\to \infty }{\lim}\left|{\tilde{e}}_{i}\right|=0,\;i\in \left\{1,2,\ldots ,n\right\}$$

**Proof.**  The tracking error (${\tilde{e}}_{i},i\in \left\{1,2,\ldots ,n\right\}$) of the closed-loop system is the error between the reference trajectory and the corresponding plant estimated states expressed as,

$${\tilde{e}}_{i}=r_{i}-{\hat{\xi }}_{i}\;,i\in \left\{1,2,\ldots ,n\right\}$$

After convergence occurs, the tracking error is described by,

(51) $${\tilde{e}}_{i}=r^{\left(i-1\right)}-\xi_{i}\;,i\in \left\{1,2,\ldots ,n\right\}$$

For the system given in (33), the states ($\xi_{i}$) are expressed in terms of the plant output, which is expressed as,

(52) $$\xi_{i}=y^{\left(i-1\right)}\;,i\in \left\{1,2,\ldots ,\rho \right\}\;$$

Substitute (52) in (51), and the tracking error is expressed by,

(53) $${\dot{\tilde{e}}}_{i}=r^{\left(i-1\right)}-y^{\left(i-1\right)}\;,i\in \left\{1,2,\ldots ,\rho \right\}$$

Differentiating the tracking error ($e_{i},\;i\in \left\{1,2,\ldots ,n\right\})\;$with regard to time yields

(54) $${\dot{\tilde{e}}}_{i}=r^{\left(i\right)}-y^{\left(i\right)}={\tilde{e}}_{i+1}\;,i\in \left\{1,2,\ldots ,n\right\}$$

It follows that the tracking error dynamics ${\tilde{e}}_{i}\;,i\in \left\{1,2,\ldots ,n\right\}$) are expressed as

(55) $$\left\{\begin{array}{c} \begin{array}{c} {\dot{\tilde{e}}}_{1}={\tilde{e}}_{2},\;\\ {\dot{\tilde{e}}}_{2}={\tilde{e}}_{3},\; \end{array}\\ \begin{array}{c} \vdots \;\\ {\dot{\tilde{e}}}_{n}=r^{\left(n\right)}-y^{\left(n\right)}=r^{\left(n\right)}-{\dot{\xi }}_{n}\; \end{array} \end{array}\right.$$

This, together with (47), yields,

(56) $$\left\{\begin{array}{c} \begin{array}{c} {\dot{\tilde{e}}}_{1}={\tilde{e}}_{2},\;\\ {\dot{\tilde{e}}}_{2}={\tilde{e}}_{3},\; \end{array}\\ \begin{array}{c} \vdots \;\\ {\dot{\tilde{e}}}_{n}=r^{\left(n\right)}-\left(f+u\right) \end{array}\; \end{array}\right.$$

From (48) and (56), we obtain,

(57) $$\left\{\begin{array}{c} \begin{array}{c} {\dot{\tilde{e}}}_{1}={\tilde{e}}_{2},\;\\ {\dot{\tilde{e}}}_{2}={\tilde{e}}_{3},\; \end{array}\\ \begin{array}{c} \vdots \;\\ {\dot{\tilde{e}}}_{n}=r^{\left(n\right)}-v+{\hat{\xi }}_{n+1}-f \end{array}\; \end{array}\right.$$

It follows from (45) and (57) that,

(58) $$\left\{\begin{array}{c} \begin{array}{c} {\dot{\tilde{e}}}_{1}={\tilde{e}}_{2},\;\\ {\dot{\tilde{e}}}_{2}={\tilde{e}}_{3},\; \end{array}\\ \begin{array}{c} \vdots \\ {\dot{\tilde{e}}}_{n}=r^{\left(n\right)}-v \end{array} \end{array}\right.$$

The tracking error dynamics given in (58) associated with the control law (v) designed in (49) produce the following dynamics

(59) $$\left\{\begin{array}{c} \begin{array}{c} {\dot{\tilde{e}}}_{1}={\tilde{e}}_{2},\;\\ {\dot{\tilde{e}}}_{2}={\tilde{e}}_{3},\; \end{array}\\ \begin{array}{c} \vdots \;\\ {\dot{\tilde{e}}}_{n}=r^{\left(n\right)}-{𝓀}_{1}\left({\tilde{e}}_{1}\right){\tilde{e}}_{1}-{𝓀}_{2}\left({\tilde{e}}_{2}\right){\tilde{e}}_{2}-\ldots -{𝓀}_{n}\left({\tilde{e}}_{n}\right){\tilde{e}}_{n} \end{array} \end{array}\right.$$

Based on assumption A6, the dynamics given in (59) can be represented in compact form as,

(60) $$\dot{\tilde{e}}=A\tilde{e}$$

where

(61) $$A=\left(\begin{array}{cc} \begin{array}{ccc} 0 & \;1\; & 0\\ 0 & 0 & 1\\ \vdots & \ldots & \ldots \end{array} & \begin{array}{ccc} \ldots & 0 & 0\\ \ldots & 0 & 0\\ \ldots & \;\vdots \; & \;\vdots \; \end{array}\\ \begin{array}{ccc} 0 & 0 & 0\\ 0 & 0 & 0\\ -{𝓀}_{1}\left({\tilde{e}}_{1}\right) & -{𝓀}_{2}\left({\tilde{e}}_{2}\right) & -{𝓀}_{3}\left({\tilde{e}}_{3}\right) \end{array} & \begin{array}{ccc} \ldots & 1 & 0\\ \ldots & 0 & 1\\ \ldots & -{𝓀}_{n-1}\left({\tilde{e}}_{n-1}\right) & -{𝓀}_{\rho }\left({\tilde{e}}_{n}\right) \end{array} \end{array}\right)$$

and $\tilde{e}=$ ${\left({\tilde{e}}_{1},{\tilde{e}}_{2},\ldots ,{\tilde{e}}_{n}\right)}^{T}$

The characteristic polynomial of A is expressed by

(62) $$\left|\lambda I-A\right|=\lambda^{n}+{𝓀}_{n}\left({\tilde{e}}_{n}\right)\lambda^{n-1}+{𝓀}_{n-1}\left({\tilde{e}}_{n-1}\right)\lambda^{n-2}+\ldots +{𝓀}_{1}\left({\tilde{e}}_{1}\right)$$

The design parameters of the proposed controller are selected to ensure that the roots of the characteristic polynomial (43) have a strictly negative real part, which makes (61) asymptotically stable. Hence, $\underset{t\to \infty }{\lim}\left|{\tilde{e}}_{i}\right|=0$. □

**Remark 1.**  *The error vector is calculated up to the relative degree (*n*) of the system because the ESO estimate system states up to* n*, i.e.,* $e_{i}=r_{i}-{\hat{\xi }}_{i}$*,* $\;i\in \left\{1,2,\ldots ,n\right\}$*. This implies that the vector* $k\left(e\right)$ *of (23) and the vector* $f\left(e\right)$ *of (24) are of size* n.

**Corollary 1.**  *Consider the nonlinear system and the control signal given in Theorem 2. The control signal (*v*) is expressed as* $v=\overset{n}{\underset{i=1}{\sum }}k_{i}\left({\tilde{e}}_{i}\right)f_{i}\left({\tilde{e}}_{i}\right)$*, where* $\;k_{i}\left({\tilde{e}}_{i}\right)=\left(k_{i1}+\frac{k_{i2}}{1+\exp\left(\mu_{i}{\tilde{e}}_{i}{}^{2}\right)}\right)$ *, and* $f_{i}\left({\tilde{e}}_{i}\right)={\left|{\tilde{e}}_{i}\right|}^{\alpha_{i}}sign\left({\tilde{e}}_{i}\right)$ *for* $i\in \left\{1,2,\ldots ,n\right\}$*. Moreover, if assumptions A5 and A6 hold, then* $\underset{t\to \infty }{\lim}\left|r_{i}-{\hat{\xi }}_{i}\right|=0,\;i\in \left\{1,2,\ldots ,\rho \right\}$ *for a suitable set of the design parameters* $k_{i1},k_{i2},\mu_{i},and\;\alpha_{i}\;$*with* $i\in \left\{1,2,\ldots ,n\right\}$.

**Proof.**  Since

(63) $$k_{i}\left({\tilde{e}}_{i}\right)f_{i}\left({\tilde{e}}_{i}\right)=\left(k_{i1}+\frac{k_{i2}}{1+\exp\left(\mu_{i}{\tilde{e}}_{i}{}^{2}\right)}\right){\left|{\tilde{e}}_{i}\right|}^{\alpha_{i}}sign\left({\tilde{e}}_{i}\right),\;i\in \left\{1,2,\ldots ,n\right\}$$

Equation (63) can be expressed as,

(64) $$k_{i}\left({\tilde{e}}_{i}\right)f_{i}\left({\tilde{e}}_{i}\right)=\left\{\begin{array}{c} \begin{array}{cc} 0\; & {\tilde{e}}_{i}=0 \end{array}\\ \begin{array}{cc} \;{𝓀}_{i}\left({\tilde{e}}_{i}\right){\tilde{e}}_{i}\; & {\tilde{e}}_{i}\neq 0 \end{array} \end{array}\right.$$

where the function ${𝓀}_{i}:\mathbb{R}/\left\{0\right\}\to {\mathbb{R}}^{+}$ is an even nonlinear gain function, and:

(65) $${𝓀}_{i}\left({\tilde{e}}_{i}\right)=\left(k_{i1}+\frac{k_{i2}}{1+\exp\left(\mu_{i}{\tilde{e}}_{i}^{2}\right)}\right){\left|{\tilde{e}}_{i}\right|}^{\alpha_{i}-1},\;i\in \left\{1,2,\ldots ,n\right\}$$

The expression (65) is time-varying because it is a function of$\;{\tilde{e}}_{i}$. For simplicity, consider that the parameters $k_{i2}=0\;\mathrm{and}\;\alpha_{i}=1$ and that the expression (65) is reduced to ${𝓀}_{i}\left({\tilde{e}}_{i}\right)=k_{i1}$. Consider the tracking error dynamics given in (59) with n=2, which provides

(66) $$\left\{\begin{array}{c} {\dot{\tilde{e}}}_{1}={\tilde{e}}_{2},\;\\ {\dot{\tilde{e}}}_{n}=-k_{11}{\tilde{e}}_{1}-k_{21}{\tilde{e}}_{2} \end{array}\right.$$

The characteristic equation of (66) is expressed as,

(67) $$\left|\lambda I-A\right|=\lambda^{2}+k_{21}\lambda +k_{11}$$

The roots of the characteristic equation (67) are $\lambda_{1,2}=-\frac{k_{21}}{2}+\frac{\sqrt{k_{21}{}^{2}-4k_{11}}}{2}$ for $k_{21}{}^{2}<4k_{11}$, which leads to a complex conjugate with a negative real part. Then, ${\tilde{e}}_{1}\to 0\;\mathrm{and}\;{\tilde{e}}_{2}\to 0$ at t→∞.

In Theorem 4, we assumed that $r^{\left(n\right)}=0$ for the case of $r^{\left(n\right)}$ in (59) not satisfying assumption A 6, i.e., $r^{\left(n\right)}\neq 0$. Then, for n=2,

(68) $$\left\{\begin{array}{c} {\dot{\tilde{e}}}_{1}={\tilde{e}}_{2},\;\\ {\dot{\tilde{e}}}_{2}=-k_{11}{\tilde{e}}_{1}-k_{21}{\tilde{e}}_{2}+r^{\left(2\right)}\left(t\right) \end{array}\right.$$

Let $q\left(t\right)=r^{\left(2\right)}\left(t\right)$ after taking the Laplace transform of both sides of (68)

$$s{\tilde{E}}_{1}\left(s\right)={\tilde{E}}_{2}\left(s\right)$$

$$s{\tilde{E}}_{2}\left(s\right)=-k_{11}{\tilde{E}}_{1}\left(s\right)-k_{21}{\tilde{E}}_{1}\left(s\right)+Q\left(s\right)$$

Solving for ${\tilde{E}}_{1}\left(s\right)$ and ${\tilde{E}}_{2}\left(s\right)$ in terms of $Q\left(s\right)$ *,* we obtain

(69) $${\tilde{E}}_{1}\left(s\right)=\frac{Q\left(s\right)}{s^{2}+k_{21}s+k_{11}}$$

(70) $${\tilde{E}}_{2}\left(s\right)=\frac{sQ\left(s\right)}{s^{2}+k_{21}s+k_{11}}$$

It can be noticed from (70) that for nonzero $r^{\left(2\right)}\left(t\right)=q\left(t\right)$, the error ${\tilde{e}}_{1}\left(t\right)$ tracks $r^{\left(2\right)}$, which means that at a steady state, ${\tilde{e}}_{1}\left(t\right)$ is nonzero, depending on $r^{\left(2\right)}\left(t\right)$. The error ${\tilde{e}}_{2}$ is the derivative of ${\tilde{e}}_{1}\left(t\right)$. □

## 4. Mismatched Disturbances

To satisfy the matched condition, the ESO assumes that the plant is expressed in the normal form [28,29]. Thus, it can only be applied to systems that can be directly expressed in the normal form or by changing variables. When a system has zero dynamics, performing such a transformation can be challenging. There are also nonlinear systems with disturbances appearing in a different channel of control input; these systems fail to satisfy the matching condition. Therefore, ADRC is no longer able to manipulate this mismatched disturbance as before. For instance, the following nonlinear model belongs to a class of uncertain nonlinear systems in a lower triangular form with mismatched disturbance [30,31,32,33,34,35],

(71) $$\left\{\begin{array}{c} \xi_{i}=a_{i}\xi_{i+1}+\phi_{i}\left(\xi_{1},\ldots ,\xi_{i}\right)+w_{i},\;i\in \left\{1,2,\ldots ,n-1\right\}\\ \xi_{n}=\phi_{n}\left(\xi_{1},\xi_{2},\ldots ,\xi_{n}\right)+w_{n}+bu,\;\\ y=\xi_{1}\; \end{array}\right.$$

where $\xi ={\left(\xi_{1}\left(t\right),\xi_{2}\left(t\right),\ldots ,\xi_{n}\left(t\right)\right)}^{T}\in {\mathbb{R}}^{n}$ is the system state, $y\left(t\right)\in \mathbb{R}$ is the measured output, $u\left(t\right)\in \mathbb{R}$ is the control input,$\;w_{i}\left(t\right)\in \mathbb{R},\;i\in \left\{1,2,\ldots ,n\right\}$ is the unknown exogenous disturbance, and b∈ℝ is the control coefficient. The function$\;\phi_{i}:{\mathbb{R}}^{i}\to \;\mathbb{R},\;i\in \left\{1,2,\ldots ,n\right\}$.

**Theorem 5.**  *A second-order nonlinear system in a lower triangular form with mismatched disturbances can be described as follows,*

(72) $$\left\{\begin{array}{c} {\dot{\xi }}_{1}=a_{1}\xi_{2}+\phi_{1}\left(\xi_{1}\right)+w_{1}\;\\ {\dot{\xi }}_{2}=\phi_{2}\left(\xi_{1},\xi_{2}\right)+w_{2}+bu\;\\ y=\xi_{1}\; \end{array}\right.$$

*where* $\xi ={\left(\xi_{1}\left(t\right),\xi_{2}\left(t\right)\right)}^{T}\in {\mathbb{R}}^{2}$ *is the system state,* $y\left(t\right)\in \mathbb{R}$ *is the measured output,* $u\left(t\right)\in \mathbb{R}$ *is the control input,*$\;w_{i}\left(t\right)\in \mathbb{R},\;i\in \left\{1,2\right\}$ *is the unknown exogenous disturbance, and* b∈ℝ *is the control coefficient. The function*$\;\phi_{i}:{\mathbb{R}}^{i}\to \;\mathbb{R},\;i\in \left\{1,2\right\}$*. If the function* $\phi_{1}$ *and the exogenous disturbance (*$w_{1}$*) are differentiable with regard to* t*, the system (72) can be transformed into the following form,*

(73) $$\left\{\begin{array}{c} {\dot{\tilde{\xi }}}_{1}={\tilde{\xi }}_{2}\;\\ {\dot{\tilde{\xi }}}_{2}=f\left({\tilde{\xi }}_{1},{\tilde{\xi }}_{2},w_{1},{\dot{w}}_{1},w_{2}\right)+\hat{b}u\\ y={\tilde{\xi }}_{1}\; \end{array}\right.\;$$

*where*$\;f\left({\tilde{\xi }}_{1},{\tilde{\xi }}_{2},w_{1},{\dot{w}}_{1},w_{2}\right)=a_{1}\phi_{2}\left({\tilde{\xi }}_{1},\frac{{\tilde{\xi }}_{2}-\phi_{1}\left({\tilde{\xi }}_{1}\right)-w_{1}}{a_{1}}\right)+\frac{\partial \phi_{1}\left({\tilde{\xi }}_{1}\right)}{\partial \xi_{1}}{\tilde{\xi }}_{2}+a_{1}w_{2}+{\dot{w}}_{1}$*, and* $\hat{b}=a_{1}b$.

**Proof.**  Let$\;{\tilde{\xi }}_{1}=\xi_{1}$ and ${\tilde{\xi }}_{2}={\dot{\xi }}_{1}$. Then,

(74) $$\;{\dot{\tilde{\xi }}}_{2}=a_{1}{\dot{\xi }}_{2}+\frac{\partial \phi_{1}\left(\xi_{1}\right)}{\partial \xi_{1}}{\dot{\xi }}_{1}+{\dot{w}}_{1}\;$$

By substituting (72) in (74), we obtain,

(75) $${\dot{\tilde{\xi }}}_{2}=a_{1}\phi_{2}\left({\tilde{\xi }}_{1},\xi_{2}\right)+\frac{\partial \phi_{1}\left({\tilde{\xi }}_{1}\right)}{\partial \xi_{1}}{\tilde{\xi }}_{2}+a_{1}w_{2}+{\dot{w}}_{1}+a_{1}bu$$

Since$\;\xi_{2}=\frac{{\tilde{\xi }}_{2}-\phi_{1}\left({\tilde{\xi }}_{1}\right)-w_{1}}{a_{1}}$, (75) can be expressed as,

(76) $${\dot{\tilde{\xi }}}_{2}=a_{1}\phi_{2}({\tilde{\xi }}_{1},\frac{{\tilde{\xi }}_{2}-\phi_{1}\left({\tilde{\xi }}_{1}\right)-w_{1}}{a_{1}})+\frac{\partial \phi_{1}\left({\tilde{\xi }}_{1}\right)}{\partial \xi_{1}}{\tilde{\xi }}_{2}+a_{1}w_{2}+{\dot{w}}_{1}+a_{1}bu$$

Finally, system (72) can be defined as,

(77) $$\left\{\begin{array}{c} {\dot{\tilde{\xi }}}_{1}={\tilde{\xi }}_{2},\;\\ {\dot{\tilde{\xi }}}_{2}=f\left({\tilde{\xi }}_{1},{\tilde{\xi }}_{2},w_{1},{\dot{w}}_{1},w_{2}\right)+\hat{b}u,\\ y={\tilde{\xi }}_{1}\; \end{array}\right.$$

where$\;f\left({\tilde{\xi }}_{1},{\tilde{\xi }}_{2},w_{1},{\dot{w}}_{1},w_{2}\right)=a_{1}\phi_{2}\left({\tilde{\xi }}_{1},\frac{{\tilde{\xi }}_{2}-\phi_{1}\left({\tilde{\xi }}_{1}\right)-w_{1}}{a_{1}}\right)+\frac{\partial \phi_{1}\left({\tilde{\xi }}_{1}\right)}{\partial \xi_{1}}{\tilde{\xi }}_{2}+a_{1}w_{2}+{\dot{w}}_{1}$, $\hat{b}=a_{1}b$. □

Theorem 5 can be generalized easily for nth-order uncertain nonlinear systems in a lower triangular form with mismatched disturbance $w_{i}\left(t\right),\;i\in \left\{1,2\ldots ,n\right\}$ as in (71).

## 5. Mathematical Modelling of The Differential Drive Mobile Robot

**Figure 8:**
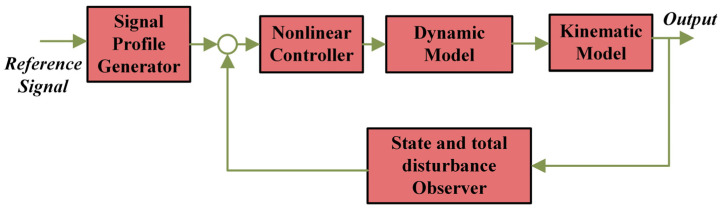
Mobile robot with an internal control loop.

The mathematical model of the mobile robot mathematical is an approximation of the physical mobile robot, which consists of the dynamical kinematic and actuator models. To restrain the robot’s motor dynamics, an internal loop is also involved. Figure 8 illustrates the mobile robot block diagram with an internal control loop [36].

As shown in Figure 8, $w\left(t\right),\;q\left(t\right)$, and $p\left(t\right)$ represent the reference input velocity, the output of the internal loop (i.e., recent velocity), and the kinematic model output (i.e., robot posture), respectively. The control inputs are the differences between the required and the recent velocities ($e\left(t\right)=w\left(t\right)-q\left(t\right)$), while the control output ($u\left(t\right)$) influences the dynamics of the mobile robot as forces or torques. The posture of the mobile robot regarding the origin of the global coordinate system (GCS) is described by the position coordinates (x,y) of its local coordinate system (LCS) origin, with rotation defined by an angle ($\theta_{m}$) [36].

**Figure 9:**
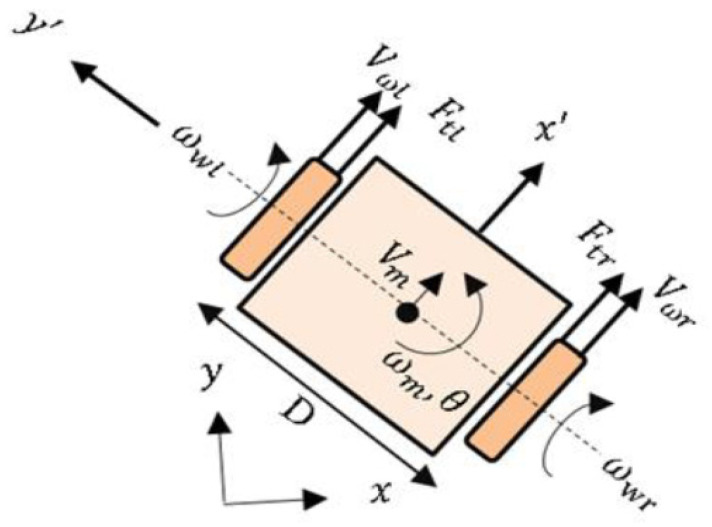
The differential drive mobile robot (DDMR).

As shown in Figure 9, the kinematic model can be described by the robot’s linear velocity ($V_{m}$) and its angular velocity ($\omega_{m}$). However, it is desirable to describe most control configurations according the wheel angular velocities ($\omega_{wr}$, $\omega_{wl}$). The general kinematic model of DDMR is defined as [37,38,39,40,41,42],

(78) $$\left\{\begin{array}{c} \dot{x}’=V_{m}cos\left(\theta_{m}\right)\\ \dot{y}’=V_{m}sin\left(\theta_{m}\right)\;\\ {\dot{\theta }}_{m}=\omega_{m} \end{array}\right.$$

Linear velocity is computed by averaging the linear velocities of the two wheels in the LCS [37,38,39,40],

(79) $$V_{m}=\frac{\left(V_{wr}+V_{wl}\right)}{2}=r_{w}\frac{\left(\omega_{wr}+\omega_{wl}\right)}{2}$$

The DDMR angular velocity is expressed as,

(80) $$\omega_{m}=\frac{\left(V_{wr}-V_{wl}\right)}{D}=r_{w}\frac{\left(\omega_{wr}-\omega_{wl}\right)}{D}\;$$

where $V_{m}$ is the longitudinal velocity of the center of mass; $\omega_{m}$ is the angular velocity of DDMR; $V_{wr}$ and$\;V_{wl}$ are the longitudinal velocities of the left and right wheels, respectively; $\omega_{wr}$ and $\omega_{wl}$ are the angular tire velocities of the left and right wheels, respectively; and $r_{w}$ is the nominal radius of the tire.

In [13], the nonlinear dynamics of the motor wheels were illustrated and presented in detail. The state-space depiction of the overall motor and wheel dynamics is summarized as follows (for the right wheel):

(81) $$J_{eq}n{\dot{\omega }}_{wr}=-B_{eq}n\omega_{wr}+k_{t}i_{ar}-{\tau^{\prime }}_{lr}$$

(82) $$L_{a}\frac{di_{ar}}{dt}=-k_{b}n\omega_{wr}-R_{a}i_{ar}+v_{ar}$$

(83) $${\tau^{\prime }}_{lr}=\tau_{rext}/n$$

where $v_{ar}$ and $v_{al}$ are the input voltages applied to the right and left motors, respectively; $i_{ar}$ and $i_{al}$ are the armature current of the right and left motors, respectively; ${\tau^{\prime }}_{lr}$ and ${\tau^{\prime }}_{ll}$ are the right and left motor-developed torques, respectively; $k_{t}$ is a torque constant; $k_{b}$ is a voltage constant; $L_{a}\;$is an electric self-inductance constant; $R_{a}\;$is an electric resistance constant; the total equivalent inertia is denoted as$\;J_{eq}$; total equivalent damping is denoted as$\;B_{eq}$; *n* is the ratio of the gearbox; and $\tau_{rext}$ and $\tau_{lext}$ are the external torque applied at the wheel side for the right land left wheels, respectively. Let $\xi_{1}=\omega_{wr}$ $\xi_{2}=i_{ar}$, $d={\tau^{\prime }}_{lr}$, and $u=v_{ar}$. Then,

(84) $${\dot{\xi }}_{1}=-\frac{B_{eq}}{J_{eq}}\xi_{1}+\frac{k_{t}}{J_{eq}n}\xi_{2}-\frac{1}{J_{eq}n}d$$

(85) $${\dot{\xi }}_{2}=-\frac{k_{b}n}{L_{a}}\xi_{1}-\frac{R_{a}}{L_{a}}\xi_{2}+\frac{1}{L_{a}}u\;$$

Let $b_{1}=-\frac{1}{J_{eq}n}$, $b_{2}=\frac{1}{L_{a}}$,

(86) $$f_{1}\left(\xi_{1},\xi_{2}\right)=-\frac{B_{eq}}{J_{eq}}\xi_{1}+\frac{k_{t}}{J_{eq}n}\xi_{2}$$

and

(87) $$f_{2}\left(\xi_{1},\xi_{2}\right)=-\frac{k_{b}n}{L_{a}}$$

The simplified model with the mismatched uncertainties and external disturbances of the DDMR exactly fits the state-space formulation given in (53). According to Theorem 1, the state-space model with mismatched uncertainties can be transformed into ADRC canonical form with $\hat{b}=\frac{1}{L_{a}}\frac{k_{t}}{J_{eq}n}$ for the motor wheel model.

## 6. Numerical Simulations

**Figure 10:**
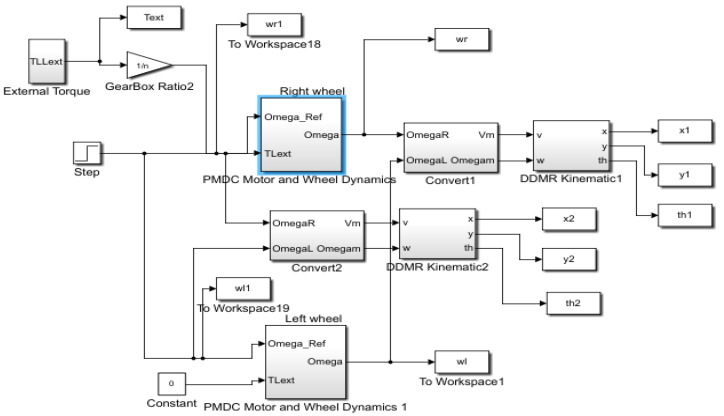
The Simulink^®^ block diagram of the DDMR kinematics and the PMDC motor controlled by the IADRC.

The kinematic model of the DDMR with PMDC motors and the proposed IADRC was designed and simulated in the MATLAB^®^/SIMULINK environment. Numerical simulations of continuous state models were conducted using the MATLAB^®^ ODE45 solver. This Runge–Kutta ODE45 solver produces a fourth-order estimate of error using a fifth-order method. Figure 10 shows the Simulink block diagram of the DDMR and the PMDC motors with IADRC.

The PMDC motor coefficient values are set to *L_a_* = 0.82, *R_a_* = 0.1557, *K_t_* = 1.1882, *K_b_* = 1.185, *B_eq_* = 0.3922, *J_eq_* = 0.2752, and *n* = 3.0. The DDMR used in the simulation is assumed to have the following coefficients: *D* = 0.40 and *r_w_* = 0.075. The coefficients of the classical ADRC controller are *δ*_1_ = 0.4620, *δ*_2_ = 0.24807, *α*_1_ = 0.1726, *α*_2_ = 0.8730, *β*_1_ = 30.4, *β*_2_ = 523.4, *β*_3_ = 2970.8, and *R* = 100. The coefficients of the proposed IADRC scheme include the coefficients of the NLSEFC, which are expressed as *k*_11_ = 144.6642, *k*_12_ = 8.0475, *k*_21_ = 25.5574, *k*_22_ = 4.8814, *k*_3_ = 0.5308, *δ* = 3.8831, *μ*_1_ = 44.3160, *μ*_2_ = 48.8179, *μ*_3_ = 26.1493, *α*_1_ = 0.9675, *α*_2_ = 1.4487, and *α*_3_ = 3.5032. The ITD suggested in this paper has a set of coefficients expressed as *α* = 0.4968, *β* = 2.1555, γ = 11.9554, and *R* = 16.8199. *K*_α_ = 0.6265, *α* = 0.8433, *K*_β_ = 0.5878, *β* = 0.04078, *β*_0_ = 30.4, *β*_1_ = 513.4, and *β*_2_ = 1570.8 represent the coefficients of the SMESO used in this work.

**Figure 11:**
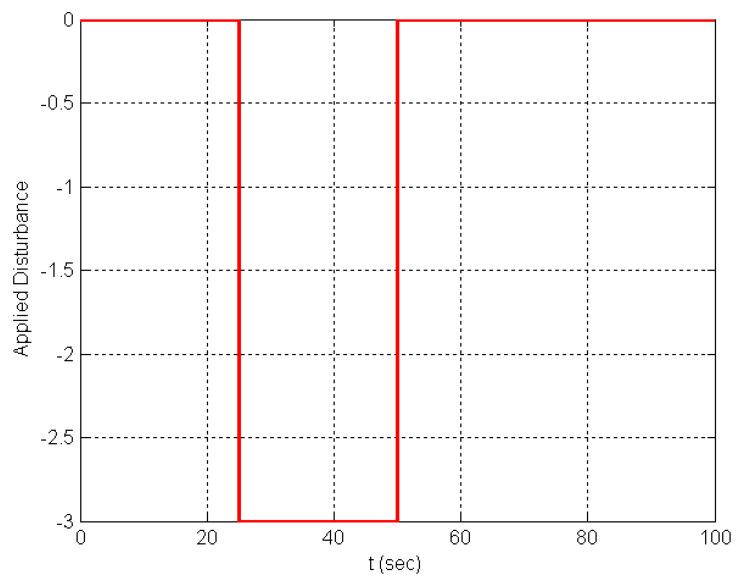
The applied external torque.

**Figure 12:**
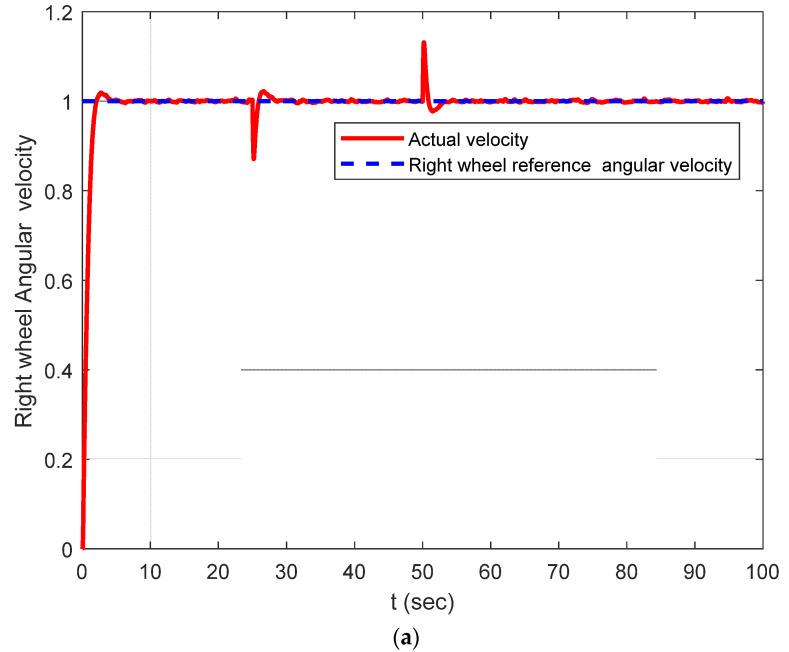

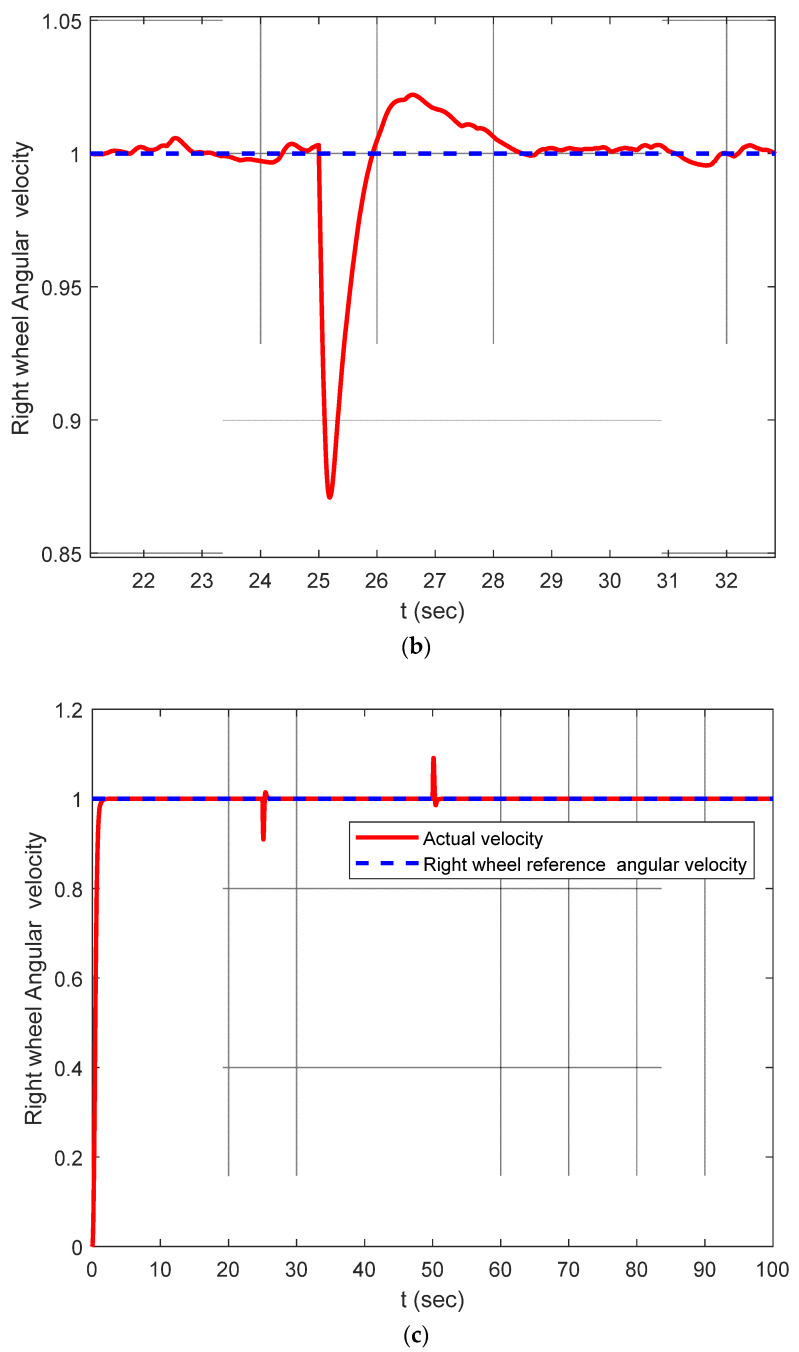

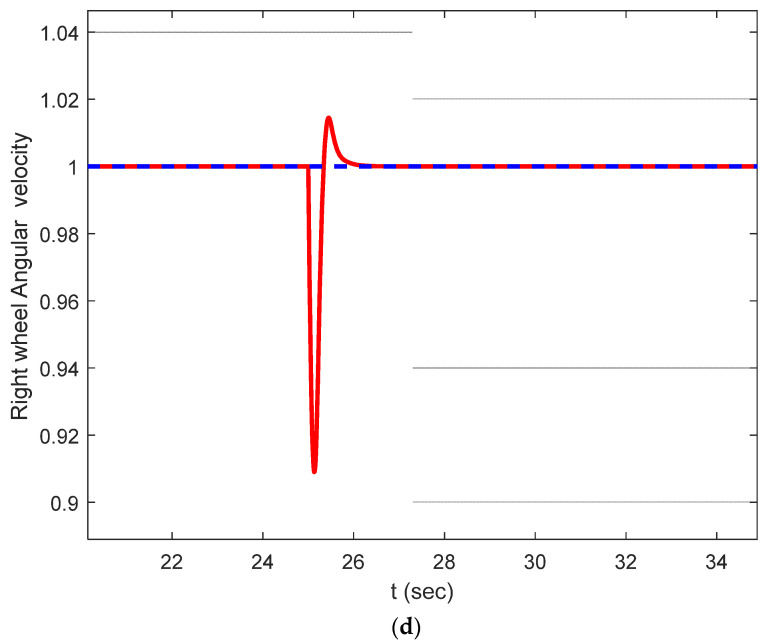
Simulation results: (**a**) the angular velocity of the right wheel using classical ADRC; (**b**) close-up of the response depicted in (**a**); (**c**) the angular velocity of the left wheel using IADRC; (**d**) close-up of the response depicted in (**c**).

The DDMR was tested by applying reference angular velocities for both wheels of 1 rad/s at t = 0 and *t* = 100 s. To examine the proposed IADRC performance, an exogenous torque acting as a constant disturbance was applied to the right wheel during the simulation at *t* = 30 and removed after 20 s. Figure 11 shows the applied external disturbance. Figure 12 shows the transient response of the controlled PMDC motor for the right wheel when both the ADRC and the IADRC are applied. The figure shows an enhancement in system response before and during the applied disturbance when the IADRC is adopted; this behavior is evident in Figure 12c,d.

**Figure 13:**
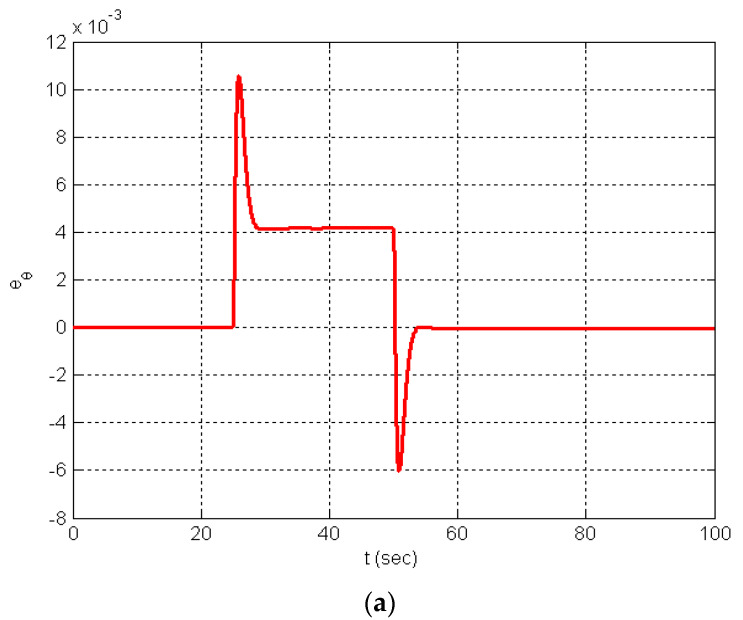

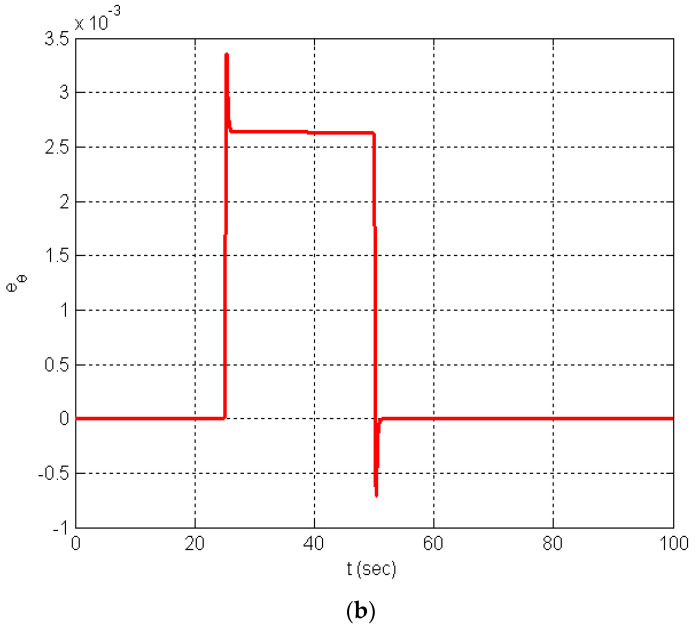
Simulation results; (**a**) the DDMR orientation error in the case of ADRC; (**b**) the DDMR orientation error in the case of IADRC.

The orientation error ($e_{\theta }$) associated with the tested case is reduced intensely due to the effectiveness of the proposed technique (see Figure 13). Note that $e_{\theta }=\theta_{ref}-\theta_{actual}$, where $\theta_{ref}$ is the orientation of the reference trajectory, and $\theta_{actual}$ is the actual orientation. The IADRC produces an error signal with less overshoot (3.4 × 10^−3^) than in the ADRC scheme (10.5 × 10^−3^). The IADRC also shows a faster convergence for the error signal because of the proposed nonlinearities in the NLSEFC controller, which strongly and quickly damp the error signals.

**Figure 14:**
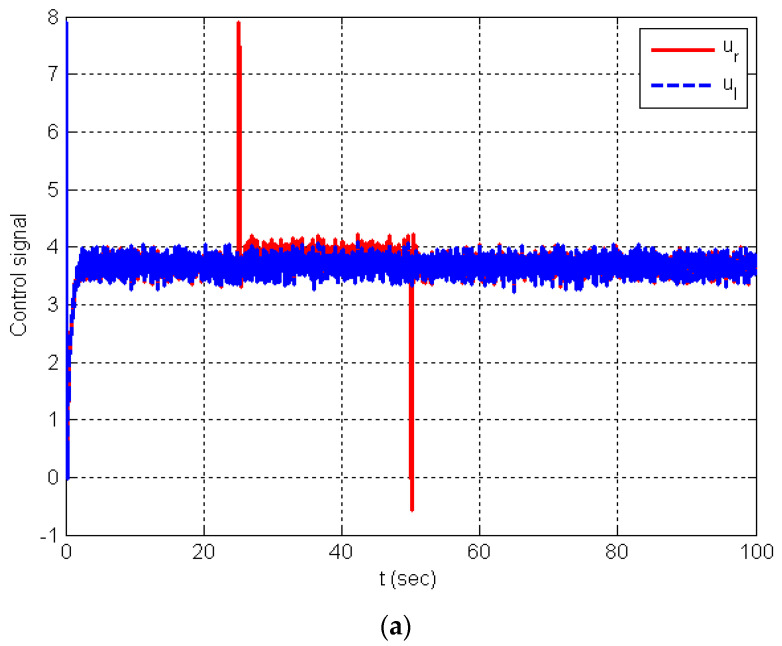

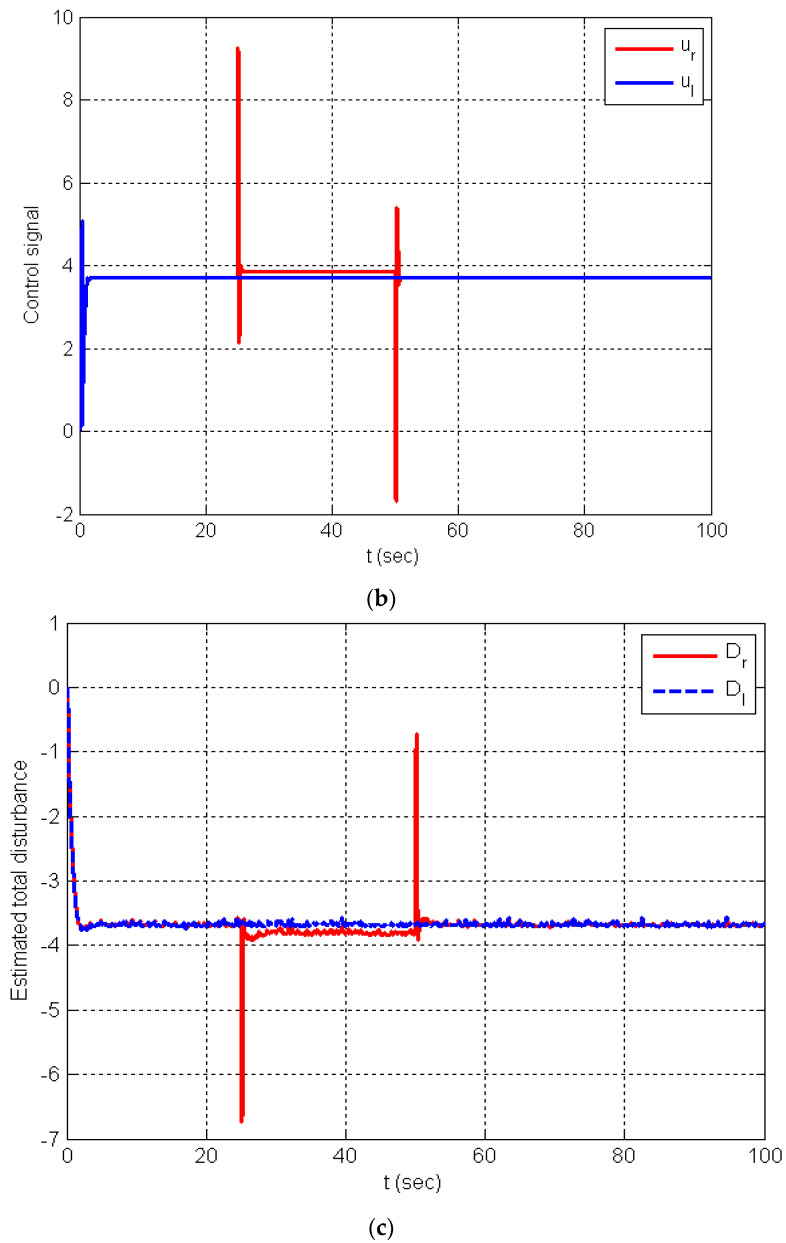

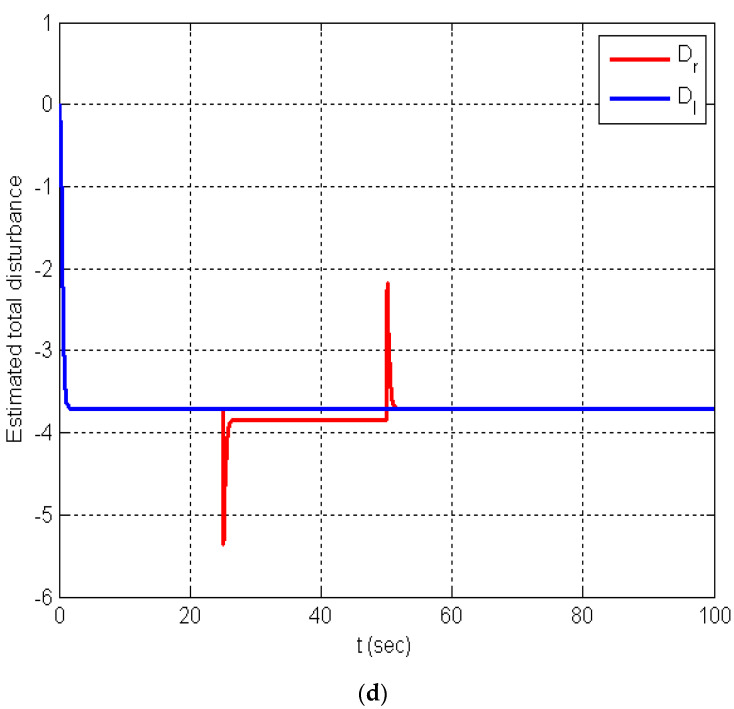
Simulation results: (**a**) the control signals generated by the ADRC; (**b**) the control signals generated by the IADRC; (**c**) the estimated total disturbance from the LESO; (**d**) the observed total disturbance from the SMESO.

The chattering phenomenon found in the estimated total disturbances produced by the LESO of the conventional ADRC for both wheels (D_r_ and D_l_) are extremely reduced by using the SMESO of the proposed IADRC. The same is true of the control signals that drive the two wheels ($u_{r}$ and $u_{l}$; see Figure 14), where a very smooth control signal is obtained as a result of the slight increase in the overshot (compare Figure 14a,b).

Table 1 and Table 2 show the results based on evaluation of several OPIs. These indices reflect the performance of the adaptive improved active disturbance rejection control. The results are classified into kinematic and dynamic performance indices.

where $OPI_{x}=\frac{1}{N}\sum {\left(x_{ref}-x_{actual}\right)}^{2}$,
$OPI_{y}=\frac{1}{N}\sum {\left(y_{ref}-y_{actual}\right)}^{2}$,
$OPI_{\theta }=\frac{1}{N}\sum {\left(\theta_{ref}-\theta_{actual}\right)}^{2}$
$ITAE=\sum t\left|\omega_{ref}-\omega_{actual}\right|dt$
$ISU=\sum u^{2}dt$

### Discussion

A new nonlinear error combination ($\Psi \left(e\right)$) is proposed, which was used to construct an NLSEFC. When used solely in the feedback loop, it leads to a noticeable improvement in the performance of the closed-loop system in terms of the ISU index for both models. This closeness is due to the common term ($f\left(e\right)={\left|e\right|}^{\alpha }sign\left(e\right)$) included in the structure of the NLSEFC. Furthermore, the nonlinear gain function ($k\left(e\right)$), in contrast to the conventional PID controller, produces a variable gain depending on the error value, which, in turn, enhances the transient behavior of the system response. Furthermore, an SMESO is suggested in this paper; the smoothness of the control signal u and the minimum overshoot in the output response are due to using the proposed nonlinear error function (·) with the following features: it is a smooth function, and it has high gain near the origin and a small gain with large error values. Finally, a new tracking differentiator, named the INTD, is proposed; it proved superior to the other tracking differentiators by solving the common issues extant in conventional differentiators. One of these issues is the “peaking phenomenon”. This phenomenon is reduced by considering the INTD of 4.35 with an optimized set of parameters, i.e., *a*_1_ and *a*_2_. In addition, the proposed INTD eliminates the “phase lag” problem that is extant in most conventional tracking differentiators due to the scaling parameters, i.e., *α* and *β*. The input scaling parameter (*α*) reduces the values of the input signal (*r*(*t*)) level (1 − *α*), while scaling parameter *β* amplifies the level of the output signal (*r*_1_(*t*)), thereby accelerating the tracking phase. When these three parts are combined to synthesize the IADRC, the proposed IADRC scheme presented in this paper and applied to DDMR achieves the improvements mentioned above in an easier manner because the nonlinear system is converted into a chain of integrators by the SMESO, which is simply a linearized system controlled by a nonlinear controller. This is reflected in the DDMR in terms of the smooth output response and chatter-free control signal. Moreover, the torque disturbance is canceled by the IADRC scheme and provides very small values for the ITAE and ISU indices, as shown in Table 1 and Table 2.

A major improvement in the kinematic indices is achieved for the IADRC against the conventional ADRC, where the $OPI_{x}$, $OPI_{y}$, and $OPI_{\theta }\;$are reduced by 51.7%, 53.78%, and 70.794%, respectively. A significant enhancement in the time-domain response is achieved (ITAE is lowered by 86.6175%) by increasing the ISU, which signifies the power provided to the PMDC motor. In addition, the chattering in the control signal caused by Han’s classical ADRC is almost eliminated by the proposed IADRC. Finally, the DDMR orientation error is clearly reduced and swiftly decreases to zero.

## 7. Conclusions

An improved nonlinear ADRC controller was developed for a DDMR to provide accurate speed tracking in the presence of high external torque disturbance. The proposed IADRC with the SMESO generates an exact estimation of the states and the total disturbance. The proposed IADRC with three parts, namely the SMESO, the NLSEF, and the INTD, provides a committed scheme to enhance the ability of the conventional ADRC to achieve disturbance estimation and attenuation. In conclusion, the simulation results show that the developed IADRC can effectively enhance the performance of the system and improve the accuracy and the speed of the PMDC motor of the DDMR under mismatched uncertainties and torque disturbance. The IADRC eliminates the chattering phenomenon, which is coherent in the conventional ADRC, with minimal increase in the overshoot of the control signal when disturbance occurs. The future directions for our proposed IADRC including extending its applications to include consensus multiagent systems. The first step will be to design a control system for every local agent for consensus disturbance rejection. The second step with involve analysis of the design for network-connected multi-input linear or nonlinear systems using relative state information of the subsystems in the neighborhood. The consensus multiagent system can be configured with in leaderless or leader–follower consensus setups under common assumptions of the network connections.

## Figures and Tables

**Table 1:** DDMR kinematic performance indices.

Performance Index	Controller	
	ADRC	IADRC
$OPI_{x}$	0.0010884970	0.0005257305
$OPI_{y}$	0.0016112239	0.0007447036
$OPI_{\theta }$	0.0000059780	0.0000017459

**Table 2:** Performance indices of both wheels.

Wheel	Performance Index	Controller	
		ADRC	IADRC
Right	ITAE	13.302889	1.780254
	ISU	1372.090423	1407.300305
Left	ITAE	6.919226	0.146694
	ISU	1343.542226	1372.124019

## Data Availability

Not applicable.

## References

[1] Åström K., Hägglund T. (2006). Advanced PID Control.

[2] Seborg D.E., Edgar T.E. (2004). Process Dynamics and Control.

[3] Han J. (1995). Extended state observer for a class of uncertain plants. Control Decis..

[4] Han J. (2009). From PID to active disturbance rejection control. IEEE Trans. Ind. Electron..

[5] Zhang C., Chen Y. (2016). Tracking Control of Ball Screw Drives Using ADRC and Equivalent-Error-Model-Based Feedforward Control. IEEE Trans. Ind. Electron..

[6] Shi M., Liu X., Shi Y. Research n Enhanced ADRC algorithm for hydraulic active suspension. Proceedings of the International Conference on Transportation, Mechanical, and Electrical Engineering (TMEE).

[7] Chenlu W., Zengqiang C., Qinglin S., Qing Z. Design of PID and ADRC based quadrotor helicopter control system. Proceedings of the Control and Decision Conference (CCDC).

[8] Wu Y., Zheng Q. (2015). ADRC or adaptive controller—A simulation study on artificial blood pump. Comput. Biol. Med..

[9] Rahman M.M., Chowdhury A.H. Comparative study of ADRC and PID based Load Frequency Control. Proceedings of the International Conference on Electrical Engineering and Information Communication Technology (ICEEICT).

[10] Ahmed A., Asad Ullah H., Haider I., Tahir U., Attique H. Analysis of Middleware and ADRC based Techniques for Networked Control. Proceedings of the 16th International Conference on Sciences and Techniques of Automatic Control & Computer Engineering.

[11] Ibraheem I.K., Abdul-Adheem W.R. (2016). On the Improved Nonlinear Tracking Differentiator based Nonlinear PID Controller Design. Int. J. Adv. Comput. Sci. Appl..

[12] Abdul-Adheem W.R., Ibraheem I.K. (2017). From PID to Nonlinear State Error Feedback Controller. Int. J. Adv. Comput. Sci. Appl..

[13] Abdul-Adheem W.R., Ibraheem I.K. (2016). Improved Sliding Mode Nonlinear Extended State Observer based Active Disturbance Rejection Control for Uncertain Systems with Unknown Total Disturbance. Int. J. Adv. Comput. Sci. Appl..

[14] Ibraheem I.K., Abdul-Adheem W.R. (2018). An Improved Active Disturbance Rejection Control for a Differential Drive Mobile Robot with Mismatched Disturbances and Uncertainties. arXiv.

[15] Huang Y., Xue W.C. (2014). Active disturbance rejection control: Methodology and theoretical analysis. ISA Trans..

[16] Guo B.Z., Zhao Z.L. (2016). Active Disturbance Rejection Control for Nonlinear Systems: An Introduction.

[17] Azar A.T., Abed A.M., Abdulmajeed F.A., Hameed I.A., Kamal N.A., Jawad A.J.M., Abbas A.H., Rashed Z.A., Hashim Z.S., Sahib M.A. (2022). A New Nonlinear Controller for the Maximum Power Point Tracking of Photovoltaic Systems in Micro Grid Applications Based on Modified Anti-Disturbance Compensation. Sustainability.

[18] Menon A.R., Mehrotra K., Mohan C., Ranka S. (1994). Characterization of a Class of Sigmoid Functions with Applications to Neural Networks. Electrical Engineering and Computer Science Technical Reports. http://surface.syr.edu/eecs_techreports/152.

[19] Khalil H.K. (1996). Nonlinear Systems.

[20] Wu S., Dong B., Ding G., Wang G., Liu G., Li Y. Backstepping sliding mode force/position control for constrained reconfigurable manipulator based on extended state observer. Proceedings of the 12th World Congress on Intelligent Control and Automation (WCICA).

[21] Yang H., Yu Y., Yuan Y., Fan X. (2015). Back-stepping control of two-link flexible manipulator based on an extended state observer. Adv. Sp. Res..

[22] Xia Y., Yang H., You X., Li H. (2014). Adaptive control for attitude synchronisation of spacecraft formation via extended state observer. IET Control Theory Appl..

[23] Lin Y.P., Lin C.L., Suebsaiprom P., Hsieh S.L. (2016). Estimating evasive acceleration for ballistic targets using an extended state observer. IEEE Trans. Aerosp. Electron. Syst..

[24] Duan H., Tian Y., Wang G. Trajectory Tracking Control of Ball and Plate System Based on Auto-Disturbance Rejection Controller. Proceedings of the 7th Asian Control Conference.

[25] Dejun L., Changjin C., Zhenxiong Z. Permanent magnet synchronous motor control system based on auto disturbances rejection controller. Proceedings of the International Conference on Mechatronic Science, Electric Engineering and Computer (MEC).

[26] Liu B., Jin Y., Chen C., Yang H. (2016). Speed Control Based on ESO for the Pitching Axis of Satellite Cameras. Math. Probl. Eng..

[27] Li J., Xia Y., Qi X., Gao Z. (2017). On the Necessity, Scheme, and Basis of the Linear-Nonlinear Switching in Active Disturbance Rejection Control. IEEE Trans. Ind. Electron..

[28] Chen Z., Xu D. (2015). Output Regulation and Active Disturbance Rejection Control: Unified Formulation and Comparison. Asian J. Control.

[29] Xue W., Huang Y. (2014). On performance analysis of ADRC for a class of MIMO lower-triangular nonlinear uncertain systems. ISA Trans..

[30] Yang J., Ding Z. (2017). Global output regulation for a class of lower triangular nonlinear systems: A feedback domination approach. Automatica.

[31] Guo B.-Z., Wu Z.-H. (2017). Output tracking for a class of nonlinear systems with mismatched uncertainties by active disturbance rejection control. Syst. Control Lett..

[32] Qian C., Li S., Frye M.T., Du H. (2012). Global finite-time stabilisation using bounded feedback for a class of non-linear systems. IET Control Theory Appl..

[33] Zhu Q. (2019). Stabilization of stochastic nonlinear delay systems with exogenous disturbances and the event-triggered feedback control. IEEE Trans. Autom. Control.

[34] Ding K., Zhu Q. (2021). Extended dissipative anti-disturbance control for delayed switched singular semi-Markovian jump systems with multi-disturbance via disturbance observer. Automatica.

[35] Yang X., Wang H., Zhu Q. (2020). Event-triggered predictive control of nonlinear stochastic systems with output delay. Automatica.

[36] Cerkala J., Jadlovska A. (2015). Nonholonomic Mobile Robot with Differential Chassis Mathematical Modelling And Implementation In Simulink with Friction in Dynamics. Acta Electrotech. Et Inform..

[37] Salem F.A. (2013). Dynamic and Kinematic Models and Control for Differential Drive Mobile Robots. Int. J. Curr. Eng. Technol..

[38] Dhaouadi R., Hatab A.A. (2013). Dynamic Modelling of Differential-Drive Mobile Robots using Lagrange and Newton-Euler Methodologies: A Unified Framework. Adv. Robot Autom..

[39] Sousa R.L.S., Forte M.D.D.N., Nogueira F.G., Torrico B.C. Trajectory Tracking Control of a Nonholonomic Mobile Robot with Differential Drive. Proceedings of the Biennial Congress of Argentina (ARGENCON).

[40] Ajeil F., Ibraheem I.K., Azar A.T., Humaidi A.J. (2020). Autonomous Navigation and Obstacle Avoidance of an Omnidirectional Mobile Robot Using Swarm Optimization and Sensors Deployment. Int. J. Adv. Robot. Syst..

[41] Ammar H.H., Azar A.T., Hassanien A., Azar A., Gaber T., Bhatnagar R.F., Tolba M. (2020). Robust Path Tracking of Mobile Robot Using Fractional Order PID Controller. Proceedings of the International Conference on Advanced Machine Learning Technologies and Applications (AMLTA2019).

[42] Zidani G., Drid S., Chrifi-Alaoui L., Benmakhlouf A., Chaouch S. Backstepping Controller for a Wheeled Mobile Robot. Proceedings of the 4th International Conference on Systems and Control.

